# Digital technologies in inpatient foodservice and nutrition care: a systematic scoping review of intake, plate waste, measurement, and implementation

**DOI:** 10.3389/fnut.2026.1951971

**Published:** 2026-09-18

**Authors:** Junxiao Yang, Jingya Tai, Chunli Dong

**Affiliations:** 1 School of Nursing, Shandong First Medical University & Shandong Academy of Medical Sciences, Jinan, China; 2 School of Nursing, Shenyang Medical College, Shenyang, China

**Keywords:** artificial intelligence, dietary intake, digital health, electronic meal ordering, hospital foodservice, implementation science, nutrition care, plate waste

## Abstract

**Background:**

Digital technologies increasingly support inpatient meal ordering, dietary intake recording, automated measurement, decision support, and foodservice workflows, but evidence across technical, service, nutritional, and clinical outcomes remains fragmented.

**Objective:**

To map digital technologies embedded in hospital foodservice and nutrition care, separate technical-validation, service process, and patient outcomes, and identify implementation and translational gaps.

**Methods:**

PubMed, Embase, Web of Science Core Collection, and Scopus were searched from inception to 30 June 2026. Two reviewers independently screened records and appraised included reports; one reviewer charted data and a second checked all entries. Findings were synthesized descriptively without meta-analysis or certainty grading.

**Results:**

Twenty-five reports published between 1996 and 2026 were included; most were single site, nonrandomized, or early stage validation studies. Technical-validation studies reported improved completeness or agreement for some digital measurement tools, but artificial-intelligence performance varied and no artificial-intelligence system had independent external validation. Service-process studies reported favorable signals for ordering flexibility, documentation, selected foodservice costs, and plate waste in some settings. Several nonrandomized comparative studies reported higher energy and/or protein intake, but digital functions were usually bundled with menu, staffing, or delivery redesign, so the independent effect of the digital component remains uncertain. One randomized trial improved nutrition documentation and care planning without changing weight or length of stay. Sustained nutritional recovery, downstream clinical outcomes, formal cost-effectiveness, and equity were rarely evaluated.

**Conclusion:**

The available evidence is most suitable for mapping technologies and proximal signals, not for firm conclusions about independent clinical effectiveness. Multicenter comparative studies should link validated measurement to documented clinical responses and evaluate generalizability, economics, and equitable access.

## 1 Introduction

Disease-related malnutrition and inadequate oral intake remain common among hospital inpatients. International nutritionDay data link reduced meal consumption with delayed discharge and higher in-hospital mortality, and the European Society for Clinical Nutrition and Metabolism guideline treats hospital nutrition as part of clinical care rather than an ancillary hospitality service (1, 2). Nutritional support for malnourished or nutritionally at risk medical inpatients improves energy and protein intake and is associated with lower mortality and nonelective readmission (3). Hospital nutrition is delivered through interconnected tasks extending from diet prescription and menu selection to meal production, delivery, consumption, documentation, and clinical response. Failures at any stage can create mismatches between prescribed diets, patient preferences, food delivered, and food consumed; delay recognition of low intake; and add avoidable waste and cost. These problems affect recovery and patient-centered care, while food production and disposal also have operational and environmental consequences (4, 5).

Digital tools span four overlapping functional levels. Patient-facing systems support menu selection, meal ordering, intake entry, and feedback (6–8). Nutrition-monitoring and decision-support tools compare recorded intake with requirements, display trends, and route referrals or care tasks (9–11). Measurement technologies use images, weights, depth sensors, radio-frequency identification, or artificial intelligence to estimate consumption, nutrient intake, and leftovers (12–15). Integrated hospital foodservice and information systems connect diet orders, production, delivery, documentation, and staff workflows (5). These groups differ in maturity and evidential meaning: an accurate measurement is not the same as a completed clinical response, and a usable interface is not evidence of improved nutritional or clinical outcomes.

Existing syntheses have concentrated mainly on electronic meal ordering. Two systematic reviews reported generally favorable signals for intake, satisfaction, plate waste, and costs, but both identified small evidence bases, heterogeneous interventions, and predominantly nonrandomized designs (16, 17). A broader review of hospital foodservice strategies considered service models, menu redesign, multidisciplinary care, and mealtime support, with digital technology forming only one component of a wider intervention landscape (4). The present review extends those syntheses by including automated intake and plate-waste measurement, nutrition dashboards and decision support, and integrated diet-safety and workflow systems. It also separates technical validity, service-process performance, patient outcomes, and implementation context instead of treating them as equivalent evidence of effectiveness.

The primary review question was which digital technologies and service-chain functions have been evaluated in inpatient foodservice and nutrition care, and what dietary, nutritional, foodservice, plate-waste, economic, and downstream clinical outcomes were reported. Secondary questions concerned measurement performance, usability, workflow integration, safety, implementation, human oversight, and equity. One broad scoping review was selected because these technologies operate across a connected measurement-response-outcome pathway and because the evidence spans heterogeneous designs and analytical units; separate focused effectiveness reviews would have fragmented the technical, service, and implementation evidence needed to understand that pathway.

## 2 Methods

### 2.1 Review design and reporting

This study was conducted as a systematic scoping review with a structured descriptive comparison. A scoping approach was selected because the evidence base comprised diverse technologies, study designs, analytical units, and outcome definitions, making evidence mapping more appropriate than estimation of a single pooled effect (18). The structured comparison was used to organize study-level findings and was not intended as a systematic review of intervention effectiveness. The review followed Joanna Briggs Institute guidance (19, 20) and PRISMA-ScR reporting (21); search reporting followed PRISMA-S (22), and study selection was documented with the PRISMA 2020 flow diagram (23). The review was not prospectively registered, and no publicly accessible protocol accompanies this report. Eligibility criteria, search concepts, and core outcome domains were specified before screening, whereas the overlapping technology categories, evidence-role labels, and pathway-based interpretation were refined during data charting and synthesis.

The primary questions were: (1) which digital technologies and service chain functions had been evaluated in inpatient foodservice and nutrition care; and (2) what dietary, nutritional, foodservice, plate-waste, economic, and downstream clinical outcomes had been reported? Secondary questions examined measurement validity and completeness, usability, workflow, safety, implementation, human oversight, and equity. These secondary domains were used to explain how technical outputs might, or might not, lead to service and patient outcomes.

### 2.2 Information sources and search strategy

Final searches were run on 30 June 2026 in PubMed, Embase.com (Elsevier), Web of Science Core Collection (Clarivate), and Scopus (Elsevier), from database inception with no lower publication date limit. Controlled vocabulary and free-text terms covered hospital inpatients and wards; foodservice, meals, menus, diet orders, dietary intake, and plate waste; and digital or electronic technologies, including artificial intelligence, image analysis, sensors, radio-frequency identification, dashboards, and decision support. Additional intake- or waste-focused combinations were used for validation studies that might not describe themselves as foodservice interventions. The search was limited to these four indexed bibliographic databases. IEEE Xplore, ACM Digital Library, CINAHL, trial registries, dissertation databases, and other gray literature sources were not searched, and no separate backward or forward citation chaining procedure was reported. The database selection emphasized indexed clinical empirical studies using actual inpatient or returned-tray data, but omission of engineering, nursing, and gray literature sources may have missed eligible early-stage or implementation reports. Complete platform specific strategies, final search dates, and database-level yields are provided in Supplementary Table S1.

### 2.3 Eligibility criteria

Eligibility was defined using the Population–Concept–Context framework. The population comprised hospital inpatients of any age. Studies using actual inpatient meals, returned trays, food components, or routine hospital foodservice data were also eligible when patient-level characteristics were not applicable. The concept required a digital technology to participate directly in at least one stage of the dietary or nutrition service pathway, including menu selection or ordering, diet order matching, meal production or delivery, dietary intake recording, plate-waste measurement, nutrition monitoring, or decision support. The context was an inpatient hospital service, including acute, tertiary, rehabilitation, specialist, and pediatric settings.

Eligible reports were full-text original empirical studies reporting at least one patient, nutritional, foodservice, waste, economic, measurement, safety, usability, workflow, or implementation outcome. Randomized and nonrandomized trials, cohort and cross-sectional studies, qualitative and mixed methods studies, validation and usability studies, implementation evaluations, and economic evaluations were eligible.

Studies were excluded when they were conducted exclusively in outpatient, community, home, residential aged-care, staff catering, or visitor catering settings; evaluated nutrition education or tele-nutrition without integration into inpatient foodservice; examined electronic screening alone without a meal, intake, or foodservice function; used only simulated or staged food without real inpatient, returned-tray, or hospital workflow data; evaluated a wholly non digital intervention; or were reviews, editorials, protocols, conference abstracts, or other nonempirical publications. Reports for which the full text could not be retrieved were recorded separately as reports not retrieved and were not assigned an eligibility decision.

### 2.4 Study selection and data charting

**Figure 1:**
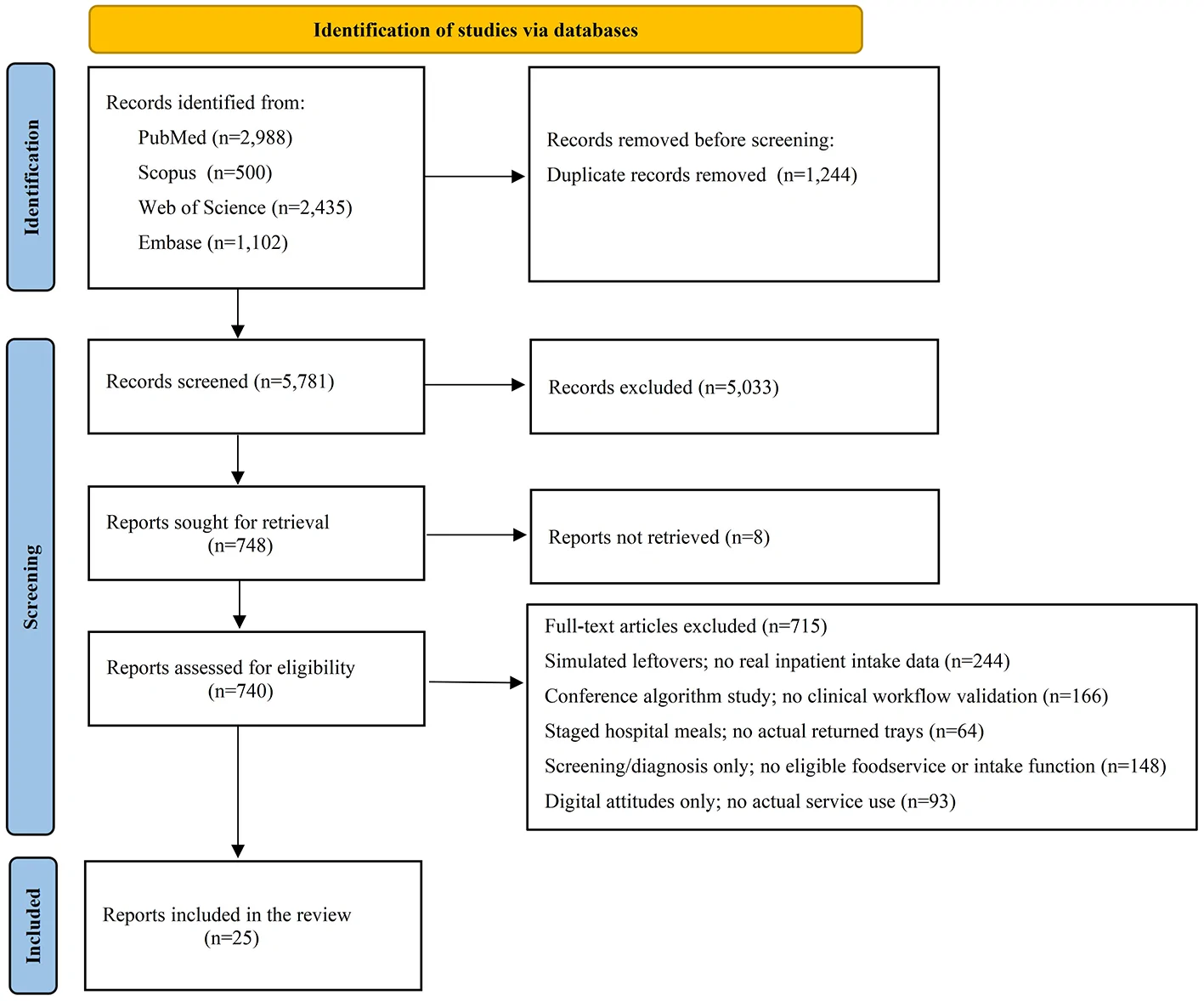
Preferred reporting items for systematic reviews and meta-analyses (PRISMA) flow diagram of report selection. The searches identified 7,025 records, of which 25 reports were included. Full-text exclusion categories were mutually exclusive, and each report was assigned one primary reason. Detailed database strategies and exclusion-category definitions are provided in Supplementary Tables S1, S2.

All retrieved records were imported into EndNote 2025 (Clarivate). Automated duplicate detection was followed by manual verification of exact and near duplicates. Two reviewers independently screened titles and abstracts and assessed potentially eligible full texts against the prespecified criteria; disagreements were resolved through discussion, with a third reviewer available when consensus could not be reached. Full-text retrieval used the access routes available to the review team at the time of the review, including database linked publisher pages, institutional holdings, and openly accessible copies. Eight reports remained unavailable through these routes and were recorded as reports not retrieved rather than being excluded on content; no eligibility judgment or direction of effect was assigned to them. A report-specific retrieval log was not retained, so undocumented author-contact or interlibrary-loan attempts are not claimed. For the 740 full texts assessed, reviewers applied the eligibility criteria and assigned one primary reason for exclusion using the mutually exclusive hierarchy shown in Figure 1 and Supplementary Table S2. The final reporting dataset retained reconciled category totals rather than a citation-level exclusion export; therefore, transparent aggregate definitions and counts are reported, and a retrospective report level list was not reconstructed without source verification.

A standardized charting form was piloted and refined before full extraction. It captured bibliographic details, country and hospital setting, study design, population or data source, sample size and analytical unit, technology and interface, primary users, data inputs and automated outputs, service chain stage, comparator or reference method, system integration and human oversight, outcomes, findings, and limitations. One reviewer charted the data and a second reviewer checked every entry against the full report. When checking identified an unclear or inconsistent entry, the reviewers returned to the source report and retained the agreed value or classification in the master charting file. Verification and corrections were made directly in that working file rather than recorded in a separate discrepancy log. Accordingly, this process is reported as second-reviewer source verification rather than independent duplicate extraction; no independently double extracted subset or inter-reviewer concordance statistic is claimed. Publications describing patient, staff, measurement, or implementation components of the same digital system were linked during synthesis so that complementary evidence was retained without treating a shared program as independent replication.

### 2.5 Methodological appraisal

Methodological limitations were appraised with design specific tools. The Joanna Briggs Institute checklists for randomized controlled trials, quasi experimental studies, analytical cross-sectional studies, qualitative research, and diagnostic test accuracy studies were applied to the corresponding designs; criterion-validation studies were assessed with the diagnostic test accuracy checklist, with nonapplicable diagnostic items recorded as such. The Mixed Methods Appraisal Tool 2018 mixed methods criteria were used for mixed methods and implementation evaluations (19, 24). Two reviewers appraised each report independently and resolved differences by consensus. Supplementary Table S3 names the tool used for every report and lists applicable checklist items judged No or Unclear.

No universal numerical quality score was calculated, and reports were not excluded solely because of methodological limitations. Appraisal findings were used explicitly to calibrate interpretation: randomized comparative evidence was given the greatest weight for patient outcomes; nonrandomized, historical, and multicomponent comparisons were described as suggestive signals; validation studies informed technical performance but not clinical effectiveness; and qualitative or implementation studies informed context and transferability. Design-specific strengths, limitations, item level No/Unclear judgments, and their role in the synthesis are presented in Supplementary Table S3.

### 2.6 Data synthesis and presentation

Findings were synthesized descriptively using tables, narrative text, and visual evidence mapping. Four overlapping categories were defined by principal function: (1) patient facing electronic menus, bedside ordering, and on demand delivery, which capture meal choices and timing; (2) digital intake recording, dashboards, and decision support, which compare intake with requirements and route feedback or care tasks; (3) image, weight, radio frequency identification, sensor, and artificial intelligence assisted measurement, which quantify intake or waste; and (4) integrated food service, diet safety, and workflow systems, which link diet orders, production, delivery, documentation, communication, or escalation. Multicomponent systems were assigned to every applicable category, and the primary evidence role was based on the function most directly linked to the reported outcome. The complete report to category mapping is provided in Supplementary Table S4B.

Outcomes were organized into dietary intake; nutritional status and nutrition-care processes; plate waste and economic outcomes; measurement validity and data completeness; downstream clinical outcomes; and service, usability, implementation, safety, and equity considerations. To avoid interpreting technical validation or contextual findings as evidence of clinical effectiveness, each report was assigned a primary evidence role: direct outcome, measurement, associational or descriptive, or contextual evidence.

Study-level estimates, measures of uncertainty, comparator characteristics, adjustment variables, and cointervention components were reproduced as reported in the source studies and organized in Supplementary Tables S4B, S4C, and S5. No standardized effect sizes were derived, and direction of effect was not used as a surrogate for certainty. For implementation, usability, safety, and equity, the charting reviewer developed descriptive, inductive codes from author-reported findings and a second reviewer checked the report-to-theme mapping; coding was not based on a predefined implementation framework. When a mapping was questioned during checking, the reviewers returned to the relevant author-reported findings and retained the agreed final category in the working synthesis table. Because these checks were incorporated directly into the final table rather than recorded in a separate thematic-discrepancy log, no thematic agreement statistic was calculated. Qualitative and quantitative evidence were integrated by juxtaposing themes with the relevant technical, service, or patient outcomes rather than by transforming or pooling them. Meta-analysis was not undertaken because technologies, designs, comparators, analytical units, and outcome definitions were substantially heterogeneous. No formal certainty grading was applied, and technology-to-outcome pathways were interpreted as plausible service mechanisms rather than causal effects unless supported by comparative evidence.

## 3 Results

### 3.1 Study selection

The searches identified 7,025 records: 2,988 from PubMed, 2,435 from Web of Science, 1,102 from Embase, and 500 from Scopus. After 1,244 duplicates were removed, 5,781 records underwent title and abstract screening, of which 5,033 were excluded. Full texts were sought for 748 reports; eight could not be obtained through the full-text access routes available to the review team and were retained in the PRISMA accounting as reports not retrieved. The remaining 740 reports were assessed for eligibility. A further 715 reports were excluded because they used simulated leftovers without real inpatient intake data (*n* = 244), reported conference algorithms without clinical workflow validation (*n* = 166), addressed screening or diagnosis without an eligible foodservice or intake function (*n* = 148), assessed digital attitudes without actual service use (*n* = 93), or used staged meals without returned-tray data (*n* = 64). Twenty-five eligible reports were included (Figure 1; Supplementary Tables S1 and S2). Figure 1 and Supplementary Table S2 provide the reconciled mutually exclusive primary reasons and aggregate counts. Because the eight unavailable reports could not be assessed, their possible eligibility and influence on the evidence map remain unknown.

### 3.2 Characteristics of the included reports

The 25 reports were published between 1996 and 2026, with 13 (52%) published from 2023 to 2026. Eleven reports were conducted in Australia, five in Denmark, two each in Norway and Japan, and one each in the United States, the Netherlands, South Korea, France, and the United Kingdom. Designs comprised seven criterion validation or comparative accuracy studies, five nonrandomized comparative or repeated service evaluations, five observational, audit, or cross sectional studies, four mixed-methods or implementation evaluations, three qualitative studies, and one randomized controlled trial. Most reports were conducted within one hospital or health service; one validation study included two tertiary hospitals. The report was retained as the counting unit because source publications did not consistently identify whether systems, institutions, or participant samples overlapped. The corpus therefore comprised 25 reports but not necessarily 25 independent technology families. Three explicit linkage clusters were identified: MyFood development and randomized evaluation (11, 25), paired patient and staff studies of the Roberts bedside electronic foodservice program (26, 27), and site/version-specific studies of the Dietary Intake Monitoring System (28, 29). These linked reports were interpreted as complementary evidence from the same program rather than independent replication. Because several reports did not name products or institutions, exact counts of unique systems, hospitals, and participant samples were not calculated to avoid false precision. Detailed characteristics and linkage information are presented in Table 1 and Supplementary Table S4A.

**Table 1:** Characteristics of included reports (*n* = 25).

Study (reference)	Country/setting	Design and sample	Digital technology	Primary outcome domain	Evidence type
van Bakel et al., (5)	Netherlands; eight hospital wards	Repeated service evaluation; 12,176 meals	Digital three-channel foodservice	Plate waste, meal frequency and cost	Direct outcome
Svendsen et al., (6)	Denmark; medical/oncology wards	Mixed-method development; questionnaire *n* = 99, usability *n* = 10	Tablet electronic ordering and meal diary	Ordering-system usability	Contextual
Terp et al., (7)	Denmark; internal medicine wards	Mixed-method pre-post feasibility; *n* = 130	Food‘n'Go ordering, intake feedback and education	Intake and requirement attainment	Direct outcome
Wray et al., (8)	United Kingdom; children's hospital	Mixed-method proof-of-concept; family/staff samples	Web-based food-on-demand ordering	Delivery feasibility and safety	Contextual
Ellick et al., (9)	Australia; digital public hospital	Implementation/quality-improvement study; service and audit data	Electronic screening, intake tracking and referrals	Malnutrition care pathway	Descriptive/direct
Maunder et al., (10)	Australia; two tertiary hospitals	Two-stage validation/feasibility; 90 trays, 45 patients/210 trays	Mobile intake electronic recording tool	Data completeness and accuracy	Measurement
Paulsen et al., (11)	Norway; university hospital	Randomized controlled trial; *n* = 100	MyFood app plus web decision support	Nutritional status and care processes	Direct outcome
Albaladejo et al., (12)	France; hospital meal service	Pilot criterion validation; 183 images/368 components	RGB-D automatic food-recognition device	Food-consumption measurement	Measurement
Liu et al., (13)	Japan; wards serving liquid diets	Comparative accuracy/usability; 174 items, 18 nurses	Automatic AI meal-tray photography	Intake/leftover measurement and usability	Measurement
Ryu et al., (14)	South Korea; inpatient setting	Prospective pilot validation; 54 enrolled, 25 analyzed	AI before/after meal imaging	Sodium-intake measurement	Measurement
Tagi et al., (15)	Japan; two hospital wards	Clinical criterion validation; 100 meals/300 components	AI-assisted liquid-food leftover estimation	Nutrient-intake measurement	Measurement
Munk et al., (30)	Denmark; university hospital wards	Cross-sectional evaluation with historical comparator; *n* = 124	Digital self-ordering and nutrition tracker	Intake adequacy and nutritional status	Direct outcome
Olufson et al., (37)	Australia; three rehabilitation units	Focused ethnography; 58 h, 165 participants	Multiple digital nutrition-care systems	Interprofessional workflow and person-centered care	Contextual
Barrington et al., (31)	Australia; oncology hospital	Comparative pre-post evaluation; *n* = 201	Bedside terminal meal ordering	Dietary intake and plate waste	Direct outcome
Barsha et al., (32)	Australia; hospital foodservices	Repeated cross-sectional comparison; *n* = 405	Four technology-supported ordering models	Foodservice satisfaction	Contextual
Jamison et al., (33)	United States; hospital ward	Comparative usability/cost evaluation; 50 recruited, 47 analyzed	Interactive computerized menu selector	Menu acceptability and cost	Contextual
Maunder et al., (34)	Australia; tertiary hospital	Quasi-experimental pre-post; *n* = 119	Electronic bedside spoken meal ordering	Energy and protein intake	Direct outcome
McCray et al., (35)	Australia; acute hospital	Retrospective pre-post audit; *n* = 188	Bedside menu ordering system	Intake, plate waste and cost	Direct outcome
Fisher et al., (36)	Australia; hospital	Retrospective validation; 216 patients, 1,783 hospital-days	Interactive nutrition dashboard	Nutritional-risk identification	Associational
Paulsen et al., (25)	Norway; university hospital	Development/criterion validation; *n* = 32	MyFood tablet intake-assessment app	Dietary-intake measurement	Measurement
Roberts et al., (26)	Australia; tertiary teaching hospital	Qualitative interviews/focus groups; *n* = 19 staff	Bedside electronic nutrition-care program	Workflow and implementation	Contextual
Roberts et al., (27)	Australia; tertiary teaching hospital	Qualitative usability study; *n* = 32 patients	Bedside electronic foodservice/nutrition system	Usability and access	Contextual
Ofei et al., (28)	Denmark; two hospital wards	Prospective meal-level study; 71 patients, 256 meals	DIMS with images, weighing and RFID	Plate waste	Direct outcome
Ofei et al., (29)	Denmark; hospital meal service	Pilot criterion validation; 17 meals	Image-weighed DIMS 2.0	Intake and plate-waste measurement	Measurement
Rattray et al., (38)	Australia; tertiary hospital	Prospective meal-process audit; 167 patients, 906 meals	Integrated electronic foodservice system	Diet safety and workflow	Contextual

The term report is used because related systems or implementation programs may be described in more than one publication. Explicit linked clusters were MyFood [Paulsen et al., (11) and (25)], the Roberts bedside electronic foodservice program (paired patient and staff reports, 2017), and the DIMS platform family [Ofei et al., (28 and (29)], evaluated as site/version-specific studies). These clusters were linked during interpretation and were not treated as independent replications of a technology. Evidence type distinguishes direct outcome, measurement, associational/descriptive, and contextual evidence; it does not represent a numerical quality grade. Detailed population characteristics, comparators, and identifiers are provided in Supplementary Table S4A. AI, artificial intelligence; DIMS, Dietary Intake Monitoring System; RFID, radio-frequency identification; RGB-D, red-green-blue plus depth imaging. Reference numbers correspond to the numbered Vancouver reference list in the main manuscript.

Adult inpatients formed the main population, including medical, surgical, oncology, rehabilitation, therapeutic-diet, liquid-diet, and nutritionally at risk groups; one proof-of-concept study was conducted in a specialist children's hospital. Across 16 reports with a principal patient-level sample, sample size ranged from 25 to 405 participants (median 109.5; interquartile range 46.5–172.3). Other reports used meals, trays, food components, hospital days, staff members, families, or qualitative observations as the analytical unit, ranging from 17 meals to 12,176 meals. Participant ages ranged from 7 to 104 years. Most assessments were cross-sectional, short-term, or conducted at discharge; one service evaluation included baseline and three annual follow-ups, and one report presented 30-day readmission and mortality data. Age was reported in 16 reports and sex in 15. Socioeconomic position, ethnicity, migration status, language, cultural background, and health literacy were not used for outcome stratification.

### 3.3 Distribution of digital technologies and service functions

**Figure 2:**
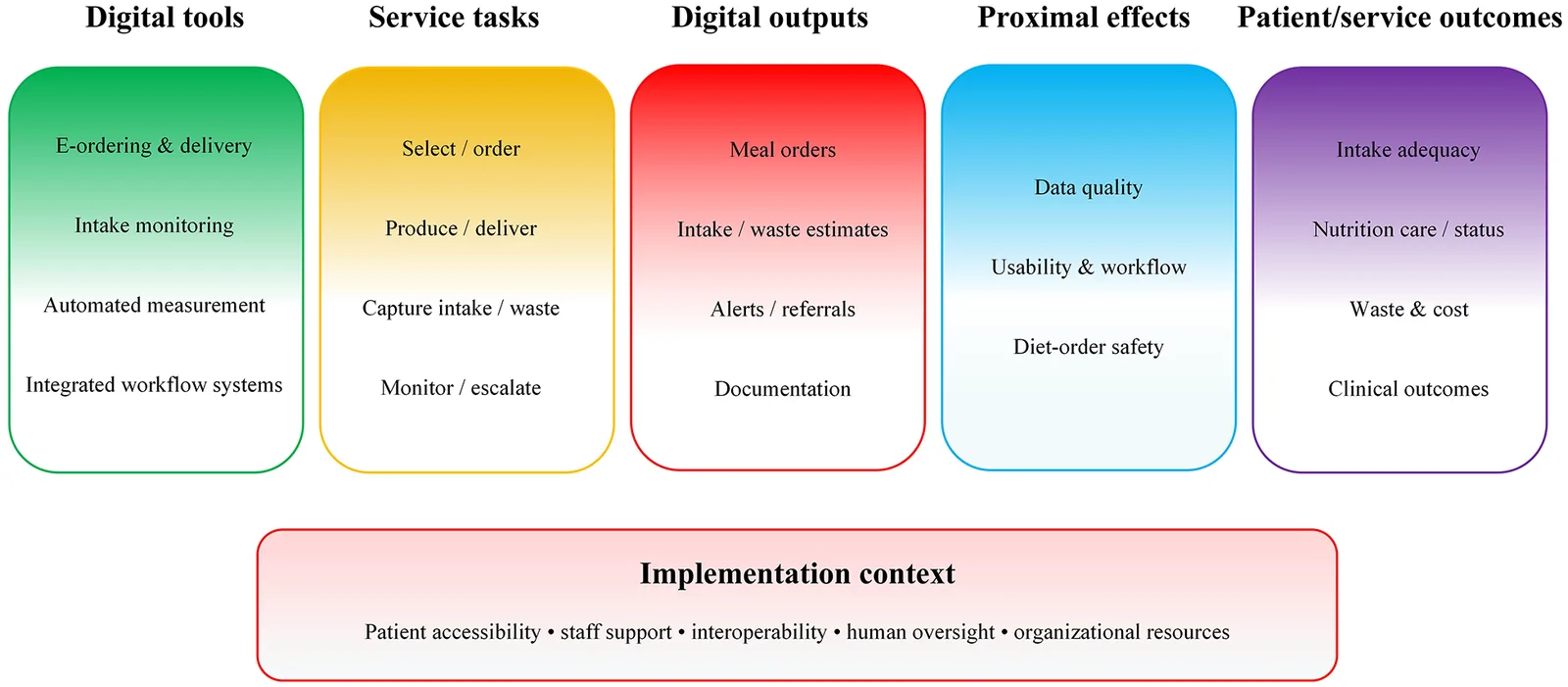
Conceptual pathway linking digital tools to inpatient foodservice and nutrition-care outcomes. Digital outputs require human verification, clinical interpretation, and an accountable response before proximal or patient/service outcomes can occur. The implementation context spans all stages.

**Figure 3:**
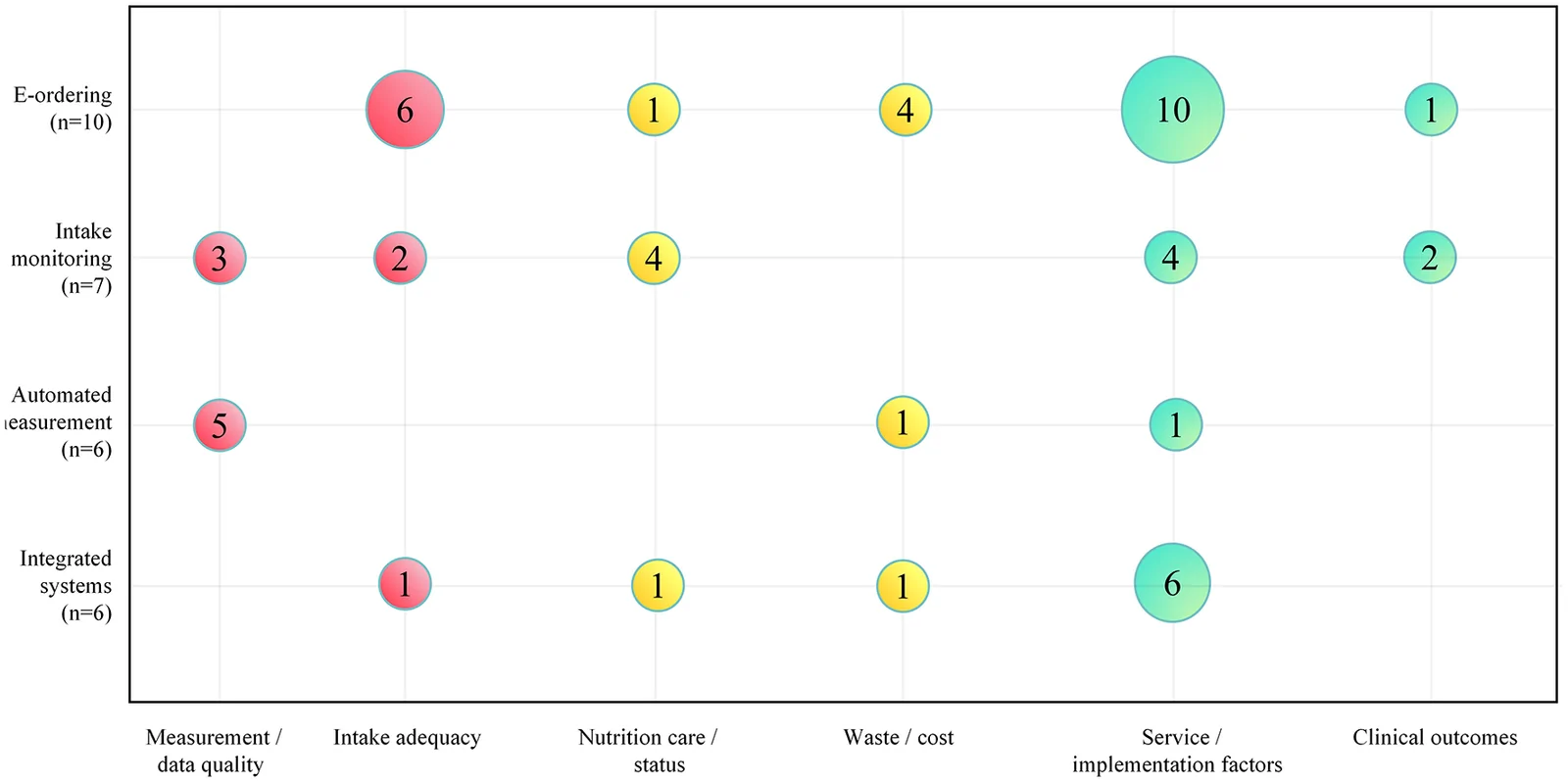
Evidence map of digital-technology categories and reported outcome domains. Bubble area is proportional to the number of contributing reports. Reports could contribute to more than one technology category and outcome domain; therefore, counts are non-mutually exclusive and do not sum to 25. Bubble color denotes the outcome-domain group, not effect direction, statistical significance, or methodological quality.

Technologies were grouped into four overlapping categories (Table 2; Figures 2, 3). Electronic menus, bedside ordering, and on-demand delivery appeared in 10 reports; digital intake recording, dashboards, and decision support in seven; image, weight, radio frequency identification, or artificial intelligence assisted measurement in six; and integrated food service, diet safety, and workflow systems in six. Functions included menu selection, order transmission, meal production and delivery, intake or plate-waste capture, nutrient calculation, intake to requirement comparison, risk visualization, automated referral, therapeutic diet checking, and staff task routing.

**Table 2:** Digital technology categories and pathways to nutrition-related outcomes.

Technology category	Evidence base	Core function and data generated	Pathway to title outcomes	Main evidence limitation
Electronic menus, bedside ordering and on-demand delivery	10 reports [e.g., (7); (30); (31); (34); (35)]	Captures meal choices and timing; supports flexible ordering, production and delivery	Reported associations with greater ordering flexibility, food availability, and energy/protein intake, and with lower waste in selected multicomponent service models	Evidence is mainly single-site and nonrandomized; technology is often bundled with menu and workflow redesign
Digital intake recording, dashboards and decision support	7 reports [e.g., (9); (10); (11); (36)]	Records intake, compares intake with requirements, visualizes trends, and routes alerts, referrals or care tasks	Provides the clearest pathway from intake data to nutrition-care action and nutritional-status surveillance	Evidence is stronger for data completeness and care processes than for weight, length of stay, readmission or mortality
Image-, weight-, RFID- and AI-assisted measurement	6 reports [e.g., (12); (13); (14); (15); (28), (29)]	Combines images, depth data, weights or tray identification to estimate intake, nutrients and leftovers	Extends objective measurement of dietary intake and plate waste and can reduce reliance on incomplete paper records	Most studies are small validation pilots with restricted food types and limited external testing
Integrated foodservice, diet-safety and workflow systems	6 reports [e.g., (9); (37); (26, 27); (38)]	Links diet orders, menu rules, staff communication, task routing, human validation and escalation	Determines whether digital foodservice data can be translated into safe, coordinated and person-centered nutrition care	Findings are predominantly contextual; interoperability, workload and local role configuration modify implementation

Reports may contribute to more than one technology category. The evidence-base column provides report counts and representative citations; the complete study-to-technology mapping is available in Supplementary Table S4B. Technology pathways describe plausible service mechanisms and should not be interpreted as causal effects unless supported by comparative outcome evidence. AI, artificial intelligence; RFID, radio-frequency identification.

Electronic ordering ranged from computerized menu selectors and spoken bedside ordering to tablet self-ordering, multichannel point of service models, and web based food on demand delivery (5–8, 30–35). Digital monitoring systems combined patient or staff- entered intake data with nutrient databases, dashboards, requirement calculations, or referral pathways (9–11, 25–27, 36). Measurement systems used pre and post meal images, tray weights, radio frequency identification, red-green-blue plus depth imaging data, or automatic photography (12–15, 28, 29). Patients, families, nurses, dietitians, nutrition assistants, physicians, and foodservice staff were users. Human review remained part of ordering, diet and allergy validation, data verification, referral, or escalation in integrated systems (8, 9, 26, 27, 37, 38).

### 3.4 Nutritional, foodservice, and downstream outcomes

Five nonrandomized comparative, pre-post, or feasibility evaluations reported favorable differences in energy and/or protein intake after digital ordering, self-ordering, or intake-feedback service changes; these findings were not treated as a vote count or as evidence of a common effect. Reported estimates included energy differences of 1,052 kJ (95% confidence interval 111–1,993) and protein differences of 17 g (95% confidence interval 926) in the Food‘n'Go pre-post study (7); 6,457 vs. 4,805 kJ and 73 vs. 58 g protein in the oncology bedside-ordering comparison (both *p* < 0.001) (31); 8,273 vs. 6,273 kJ and 83 vs. 66 g protein with spoken electronic ordering (both *p* < 0.05) (34); and 6,232 vs. 5,513 kJ (*p* = 0.035) and 78 vs.53 g protein (*p* < 0.001) with the Bedside Menu Ordering System (35). A historical comparison of the Digital Delights service reported that 70% vs. 35% met at least 75% of energy requirements and 51% vs. 24% met at least 75% of protein requirements (both *p* < 0.001) (30). Study designs, sample sizes, comparators, and cointerventions are detailed in Supplementary Tables S4B, S5. Because the interventions also changed menus, food availability, delivery, education, or staff support, the independent contribution of the digital component could not be isolated.

In the MyFood randomized trial, nutritional risk at discharge was reported in 77% of the intervention group and 94% of controls; nutritional treatment was documented in 81% vs. 57%, and a nutrition care plan in 70% vs. 16%. Weight change and length of stay did not differ between groups (11). The highest vs. lowest Nutrition Dashboard risk category was associated with Malnutrition Screening Tool risk (odds ratio 1.93, 95% confidence interval 1.17–3.19), although lower body weight was the only significant predictor after weight was added to the model (36). In a digital-hospital implementation, dietetic assistants completed 60% of inpatient dietetic occasions and 26% of assistant tasks followed nursing screening; a hospital-wide audit reported 14% malnutrition, no hospital-acquired malnutrition, and no clinical incidents (9). The digital food-concept cohort reported incomplete screening and intake documentation, and no significant differences in length of stay, 30-day readmission, or mortality by nutritional-risk group (30).

#### 3.4.1 Plate-waste findings varied by method and service model

Larger portions were associated with more waste in the Dietary Intake Monitoring System meal-level study (β = 0.008, standard error = 0.003, *p* = 0.002), and patients at nutritional risk generated more supper waste (28). Waste did not change after bedside terminal ordering in oncology (31), whereas it decreased from 30% to 26% with the Bedside Menu Ordering System, alongside a reported 19% reduction in patient food cost (35). In a three-channel digital foodservice evaluation, waste decreased from 81 g to 33–49 g per served meal and estimated production and disposal costs were 32%-56% lower across follow-up assessments (5). A computerized menu study estimated monthly costs of US$615 vs. US$2,093 for printed menus (33). Across four ordering models, adjusted satisfaction scores ranged from 3.82 for traditional menus to 4.51 for room service (32).

### 3.5 Measurement performance and validation profile

Eight reports contributed validity, accuracy, completeness, or usability findings; seven had measurement or comparative accuracy as their primary design (Supplementary Tables S3, S5). In the MyFood development study, approximately half of participants achieved at least 90% agreement with the photographic and partial-weighing reference for energy, protein, and fluid on both recording days (25). The image-weighed Dietary Intake Monitoring System reported correlations of r = 0.99 for energy and r = 0.98 for protein, with intraclass correlation coefficients of 0.88 for pre-meal portion estimates and 0.99 for post-meal portions and nutrient estimates (29). Mobile Intake did not differ from weighed food records for energy in stage 1; median recording time was 40.3 sec with Mobile Intake, 174.1 sec with paper food-record charts, and 371.4 sec with weighed records. In stage 2, incomplete records occurred in 2% vs. 65%, and full-day data were available in 93% vs. 33%, for Mobile Intake and paper charts, respectively (10). Inter-rater agreement for Nutrition Dashboard intake coding was kappa = 0.69 (95% confidence interval 0.65–0.72) (36). These findings describe technical agreement, efficiency, or completeness and do not by themselves establish that the output was clinically adequate or improved patient outcomes.

Four reports used artificial-intelligence-assisted image analysis, for liquid-food estimation, energy root-mean-square errors were 8.12 for artificial intelligence, 8.49 for image-based visual assessment, and 4.34 for direct visual assessment; the artificial intelligence estimate did not differ from weighing (*p* = 0.82), and nutrient correlations ranged from rho = 0.89 to 0.97 (15). In a sodium-imaging pilot, 25 of 54 enrolled participants were analyzed; adjusted artificial-intelligence-estimated sodium intake was 2,022.7 mg/day compared with 2,783 mg/day from 24 h urinary sodium (14). The red-green-blue plus depth prototype reported concordance correlation coefficients of 0.957 for cereals and starches, 0.845 for meat and fish, and 0.767 for vegetables (12). For automatic meal tray photography, mean absolute error was 0.18 with visual assessment, 0.80 with artificial intelligence analysis of manually captured images, and 1.72 with automatic capture; corresponding root mean square errors were 0.56, 1.65, and 2.99 (13). High correlation or concordance was interpreted alongside absolute error, attrition, missingness, food category performance, and capture failures. Validation was predominantly conducted in the development hospital or meal service setting, and no artificial-intelligence-assisted report tested performance in an independent hospital population; clinical decision adequacy and patient benefit therefore remain unestablished.

### 3.6 Clinical integration, usability, and human-context evidence

Patient-facing reports examined preference, self ordering capability, interface use, and perceived participation. In the computerized menu study, 73%-80% of respondents preferred the electronic format across measures of interest, convenience, availability, satisfaction, or motivation (33). A qualitative usability study identified familiarity with technology, interface design, perceived benefit, variation in participation, and the perceived importance of nutrition as influences on use (27). In the MyFood development study, 90% reported that the application was easy to use and 97% that it was easy to navigate (25). For the Danish ordering prototype, 78.7% considered the menu appropriate and 79.4% believed they could order independently; the System Usability Scale increased from 59.2 to 68.1 after redesign (6). Most Food'n'Go participants could use the ordering function, whereas independent intake registration was less frequent (7). Families and staff in the pediatric proof-of-concept reported favorable acceptability but identified communication, ward validation, kitchen logistics, and delivery reliability as operational requirements (8).

Staff-focused and ethnographic evidence described role allocation, communication, and safety. Staff using a bedside nutrition program emphasized patient participation, nutrition-care optimization, role clarity, workflow fit, and support requirements (26). Across three rehabilitation units, digital systems increased access to information in some settings but also made nutrition-related work less visible or created professional and system silos (37). In a therapeutic-diet audit, 69 errors were identified among 906 meals and 97% were classified as critical; the highest step specific rate occurred during main-meal delivery (38). Automated referral and delegated dietetic assistant pathways were embedded in the digital hospital implementation, while ward staff manually validated diet and allergy information before pediatric food on demand orders were released (8, 9).

Electronic health record or foodservice system integration was reported inconsistently, and several platforms were standalone or only partially connected. No study compared implementation across countries or cultural groups, and none stratified uptake or outcomes by socioeconomic position, ethnicity, migration status, language, or health literacy. Several usability studies excluded patients who were acutely unwell, cognitively impaired, isolated, or unable to participate independently; other systems retained assisted ordering, staff-entered data, telephone ordering, or family involvement. Formal health-economic evaluation beyond service cost estimates and food-waste calculations was not reported. Implementation, workflow, safety, and equity findings are summarized in Supplementary Table S6.

### 3.7 Methodological appraisal

The appraisal reflected the design profile of the evidence base. The randomized MyFood trial provided the strongest comparative evidence for patient and care process outcomes, but it tested a multicomponent decision-support pathway and found no improvement in weight or length of stay. Nonrandomized comparisons frequently used historical or service level comparators, short follow-up, and concurrent menu or workflow changes, so their intake, waste, and cost findings were interpreted as favorable signals rather than confirmatory effects. Validation studies were usually small, conducted in the development setting, and limited in food type or external transportability; they were used to characterize technical performance only. Qualitative and implementation reports offered detailed local context but were predominantly single-site and did not establish effectiveness. No report was excluded on the basis of appraisal; item-level No/Unclear judgments and their interpretive consequences are summarized in Supplementary Table S3.

## 4 Discussion

### 4.1 Principal interpretation

Interpretation was organized around a measurement response outcome pathway. At the measurement stage, several tools improved data completeness or reported agreement with reference methods, although external validation and clinically acceptable error thresholds were limited. At the response stage, digital systems improved ordering flexibility, documentation, referrals, or care planning in selected settings, but responsibility, workflow integration, and delivery of the resulting intervention were inconsistently measured. At the outcome stage, favorable intake or foodservice signals came mainly from nonrandomized multicomponent evaluations, while sustained nutritional recovery and downstream clinical benefit were rarely demonstrated. The strength of inference therefore decreases from technical performance to service process response and then to patient outcomes. A valid measurement or usable interface may be necessary for better care, but it is not sufficient unless data are reviewed, linked to a feasible intervention, and followed to an outcome (11, 39, 40).

This interpretation is consistent with the MyFood trial, in which documentation and care planning improved but weight and length of stay did not change during a relatively short admission (11). Such findings place process outcomes in context rather than dismissing them. Improved visibility of low intake may be a necessary precursor to treatment, but it is not evidence that treatment was delivered or sustained. Disease severity, procedures, symptoms, length of exposure, and post-discharge care also influence distal outcomes (4).

### 4.2 Interpreting reported signals from electronic ordering

Electronic ordering contributed the largest cluster of comparative intake and service evaluations in this review, but the evidence remained predominantly nonrandomized and multicomponent. A plausible service pathway is that ordering closer to consumption may reduce mismatch between earlier choices and current appetite, while greater menu visibility and flexible delivery may improve access to acceptable food. Comparisons among ordering models suggest that timing and flexibility may contribute to satisfaction independently of the interface, although these components were not experimentally isolated (32).

Attribution remains difficult because electronic ordering was often introduced with a la carte menus, energy- and protein-dense foods, education, additional eating opportunities, or staff assistance. The observed findings are therefore best understood as signals from redesigned service models enabled by technology, not as isolated effects of replacing paper with a screen. This limits causal separation but reflects routine foodservice, where an interface has little capacity to improve intake if production, delivery, menu design, and bedside support remain unchanged (7, 30). Accordingly, the intervention is better conceptualized as a sociotechnical service configuration whose adoption and effects depend on technology, users, professional roles, workflow, organizational capacity, and perceived value (41–43). Recent outpatient evidence likewise links technology supported hospital services to patient experience through structural conditions and care processes rather than treating technology as a standalone determinant; its setting and outcome differ from inpatient nutrition care, so it provides conceptual rather than direct effectiveness support (44).

Plate waste requires a similarly cautious interpretation. Waste is shaped by portion size, food provided, appetite, preference, disease burden, and production practice; it is not simply the inverse of intake. A reduction may indicate better alignment of supply with demand, but it may also follow smaller portions or less food provision. Evaluations therefore need to report intake adequacy, food provided, waste, patient experience, and cost together. Personalized and on demand models may align these outcomes, but current economic evidence remains limited to service cost and waste estimates rather than full cost effectiveness (5).

### 4.3 From measurement performance to clinical action

Digital recording and automated measurement address a persistent weakness in inpatient nutrition care: intake is often incompletely documented or recognized too late. Technical accuracy alone, however, does not improve care when meals are missed, data are not integrated, alerts are not reviewed, or no intervention follows. Performance also varied by food type, presentation, and image capture method, and none of the artificial intelligence systems was validated in an independent hospital population (12–15). The clinically relevant standard is therefore not correlation alone, but whether absolute error, systematic bias, missingness, and failure rates are small enough to support a safe decision in routine practice. For method comparison studies, high correlation should not be treated as evidence of interchangeability; agreement, systematic bias, limits of agreement, missingness, and failure cases are more clinically informative (45). Early stage clinical evaluations of artificial-intelligence systems should also report human factors, workflow integration, safety, and performance under routine use (46).

The gap between improved processes and downstream outcomes points to the response pathway as a major source of uncertainty. Future evaluations can make this pathway measurable by recording who reviewed a low intake signal, the time to review, the action selected, whether the intervention was delivered, and whether intake subsequently improved. These measures would show where a system fails and whether poor outcomes arise from measurement error, delayed review, unclear ownership, or limited treatment capacity (11).

### 4.4 Implementation, safety, and equitable access

Digitalization redistributes nutrition-related work rather than simply removing it. Better data visibility can improve coordination, but poor interoperability may duplicate documentation, shift tasks without agreement, or create professional silos. Nurses frequently sit at the junction of screening, meal observation, intake documentation, patient assistance, and escalation. A technically sound system may therefore have little effect when responsibility for reviewing and acting on its output is unclear. Explicit role allocation, shared access to relevant information, protocolized escalation, and dietitian oversight are central implementation requirements (9, 37). Implementation outcomes should be specified separately from service and clinical outcomes, including acceptability, adoption, appropriateness, feasibility, fidelity, implementation cost, penetration, and sustainability (47). Interoperability is not merely a technical feature; it is a precondition for reliable information flow across foodservice, nursing, dietetic, and medical systems (48).

Human verification remains a safety control. Electronic diet rules and ordering platforms cannot eliminate errors during tray preparation, ward delivery, or consumption of patient-sourced food. Digital systems may reduce transcription and communication failures, but therapeutic-diet and allergy management still requires accountable review at critical points in the pathway (38).

Usability findings may overstate accessibility because patients who were acutely unwell, cognitively impaired, unfamiliar with digital technology, or functionally dependent were often underrepresented. These groups may also have the greatest nutritional risk. Assisted ordering, staff entered data, family participation, telephone ordering, and non digital alternatives are therefore core service features rather than temporary exceptions (27). The absence of subgroup specific reporting indicates underrepresentation and a plausible risk of unequal access or benefit; it does not demonstrate that digital interventions caused inequitable outcomes (49).

### 4.5 Research priorities

The next phase of research needs to evaluate complete pathways rather than treating technical performance, adoption, and patient outcomes as interchangeable. Multicenter pragmatic trials and hybrid effectiveness implementation designs can evaluate patient benefit and implementation performance within the same study (50). When digital systems are introduced alongside changes in menus, staffing, production, or delivery, embedded process evaluation should test fidelity, mechanisms, contextual contingencies, and interactions among components rather than treating the package as a single black-box intervention (51, 52).

Measurement technologies require independent validation across hospitals, culturally diverse menus, mixed dishes, modified texture diets, and routine workflows. Reports need absolute error, systematic bias, failure rates, missingness, and clinically acceptable thresholds, not correlation alone. Economic evaluation should include acquisition, integration, maintenance, training, staff time, food production, and disposal, and should follow current reporting guidance (53). Plate waste studies should apply standardized audit methods so that estimates can be compared across institutions and over time (54). Equity analyses should report access, assistance requirements, safety, and outcomes by age, cognitive status, language, digital confidence, and functional dependency. These priorities are summarized in Table 3.

**Table 3:** Cross-domain evidence synthesis and priority research gaps.

Domain	Condensed evidence summary	Overall interpretation	Principal limitation	Priority research need
Dietary intake	Five nonrandomized comparative, pre-post, historical, or feasibility reports described favorable energy and/or protein differences after digitally enabled service changes	Favorable report-level signals; not a pooled estimate or proof of an independent digital effect	Single-site, short-term, and multicomponent evaluations; effect magnitude, precision, comparator type, and cointerventions varied	Use multicentre controlled designs, isolate the digital component and assess sustained intake adequacy in high-risk groups
Nutritional status and nutrition care	One randomized trial improved documentation and nutritional-risk status at discharge without improving weight or length of stay; other studies mainly assessed screening, risk identification and care processes	Process outcomes are better supported than patient-level nutritional recovery	Few longitudinal measurements of weight change, malnutrition resolution or documented responses to digital alerts	Prespecify nutritional-status endpoints and link alerts to verified clinical actions and follow-up
Plate waste and economic outcomes	Two service evaluations reported lower waste after digital foodservice redesign, one reported no material change, and one linked portion size and nutritional risk to waste	Potential benefit, with inconsistent findings	Waste definitions, observation periods and economic assumptions varied; concurrent controls were uncommon	Standardize gram- and percentage-based waste metrics, use full cost accounting and extend follow-up
Measurement validity and data completeness	Image-weighed and electronic tools often improved completeness or agreement relative to paper records, whereas AI accuracy varied by food type and capture method	Promising measurement support; not equivalent to demonstrated clinical effectiveness	Small single-site samples, clustered observations, restricted menus and little external validation	Define clinically acceptable error thresholds and validate tools in routine mixed-meal workflows across hospitals
Downstream clinical outcomes and equity	Weight, length of stay, readmission, mortality, complications and subgroup-specific access were rarely evaluated; beneficial clinical effects were not demonstrated	Evidence is insufficient	Most studies prioritized feasibility, service performance or technical validation and often excluded patients with greater support needs	Conduct pragmatic trials that retain assisted/non-digital options and report uptake, safety and outcomes by age, cognition, language and dependency

This table maps report-level evidence patterns and key limitations; it does not constitute vote counting, a pooled effect estimate, or certainty grading. Direction summaries do not give equal evidentiary weight to studies and should be interpreted with design, sample size, precision, comparator type, cointerventions, and methodological appraisal. Design-specific considerations are presented in Supplementary Table S3; full study-level quantitative and qualitative findings are reported in Supplementary Tables S4C, S5; and implementation, workflow, and equity considerations are summarized in Supplementary Table S6. No pooled effect estimates were calculated because of substantial heterogeneity in interventions, designs, units of analysis, and outcome definitions. AI, artificial intelligence.

### 4.6 Strengths and limitations

A principal strength of this review is its pathway-level synthesis of patient-facing ordering, intake monitoring and decision support, automated measurement, and integrated workflow systems. Separating technical validation, service process outcomes, patient outcomes, and contextual evidence reduced the risk of interpreting measurement performance as clinical effectiveness. The evidence base nevertheless spans three decades of rapidly changing technologies, heterogeneous comparators and analytical units, and highly variable outcome definitions. Most evaluations were single-site and nonrandomized, and digital functions were frequently bundled with changes to menus, staffing, production, or delivery. Effect magnitude and precision could not be standardized, meta-analysis was not appropriate, and the independent contribution of the digital component could not be estimated.

The review was not prospectively registered and no public protocol accompanies this report, which limits assessment of whether all synthesis decisions were established *a priori*. The search was restricted to four bibliographic databases and did not include IEEE Xplore, ACM Digital Library, CINAHL, trial registries, dissertation databases, other gray literature sources, or a separate citation-chaining procedure; full text original empirical publication criteria also reduced coverage of early technical reports. Eight reports could not be retrieved through the full-text access routes available to the review team and therefore could not be assessed for eligibility; report specific retrieval steps were not retained in a separate log. Full-text exclusions are reported as reconciled mutually exclusive aggregate categories because a citation-level export was not retained in the final reporting dataset. Data were charted by one reviewer and checked against the source reports by a second reviewer, with questions resolved in the working charting file; this was not independent duplicate extraction, and correction counts or concordance statistics were not available. Similarly, inductive thematic mappings were checked and finalized in the working synthesis table without a separate thematic-discrepancy log. The report was used as the counting unit, and known publication clusters were linked, but exact counts of unique systems, hospitals, and participant samples could not be derived without assumptions. These documentation and design features reduce auditability and protection against classification error but are reported transparently so that the scope of the evidence map is not overstated. Technology categories, evidence-role assignments, and inductive implementation and equity themes necessarily involved reviewer judgment. Finally, favorable direction summaries were descriptive and did not account uniformly for sample size, precision, comparator type, cointerventions, or risk of bias; they should not be interpreted as certainty or as a quantitative estimate of effectiveness. Publication bias and selective outcome reporting could not be formally assessed.

## 5 Conclusion

Available evidence shows favorable signals for meal ordering, intake recording, data completeness, nutrition care documentation, and selected foodservice processes. It does not establish that digital technologies independently improve sustained nutritional recovery or downstream clinical outcomes, because most comparative studies were single-site, nonrandomized, and multicomponent.

The conclusion is therefore an evidence map rather than an estimate of efficacy. Independent effectiveness, generalizability, cost effectiveness, safety, and equity require further evaluation through externally validated measurement, multicenter comparative studies, and hybrid effectiveness implementation designs that document who reviews a digital signal, what response follows, and whether patient outcomes improve.

## Data Availability

The raw data supporting the conclusions of this article will be made available by the authors, without undue reservation.
