# Supplementary material for: Digital technologies in inpatient foodservice and nutrition care: a systematic scoping review of intake, plate waste, measurement, and implementation
Source: Front Nutr. 2026 Sep 18;13:1951971. doi: 10.3389/fnut.2026.1951971 (PMC13589689; doi:10.3389/fnut.2026.1951971)
Supplement: Supplementary file 1 [file Table_1.DOCX]

**Supplementary Table S1. Database search strategies**

| **Database** | **Final search date** | **Complete search strategy** | **Records retrieved** |
| --- | --- | --- | --- |
| PubMed/MEDLINE | 30 June 2026 | ( "Inpatients"[Mesh] OR "Hospitals"[Mesh] OR "Hospitalization"[Mesh] OR inpatient*[tiab] OR hospital*[tiab] OR hospitalized[tiab] OR hospitalised[tiab] OR ward*[tiab] OR "acute care"[tiab] OR "hospital patient*"[tiab] OR "admitted patient*"[tiab]) AND ((( "Food Service, Hospital"[Mesh] OR "Food Services"[Mesh] OR "Dietary Services"[Mesh] OR "Menu Planning"[Mesh] OR "Dietetics"[Mesh] OR foodservice*[tiab] OR "food service*"[tiab] OR "dietary service*"[tiab] OR "nutrition service*"[tiab] OR "nutrition care"[tiab] OR "nutritional care"[tiab] OR meal*[tiab] OR menu*[tiab] OR catering[tiab] OR "hospital food*"[tiab] OR "hospital diet*"[tiab] OR "patient meal*"[tiab] OR "patient food*"[tiab] OR "meal tray*"[tiab] OR "food tray*"[tiab] OR "meal deliver*"[tiab] OR "food deliver*"[tiab] OR "meal order*"[tiab] OR "food order*"[tiab] OR "diet order*"[tiab] OR "therapeutic diet*"[tiab]) AND ( "Medical Informatics Applications"[Mesh] OR "Hospital Information Systems"[Mesh] OR "Point-of- Care Systems"[Mesh] OR "Mobile Applications"[Mesh] OR "Artificial Intelligence"[Mesh] OR "Machine Learning"[Mesh] OR "Image Processing, Computer-Assisted"[Mesh] OR digital*[tiab] OR electronic*[tiab] OR computer*[tiab] OR technolog*[tiab] OR informat*[tiab] OR software[tiab] OR automation[tiab] OR automated[tiab] OR automatic[tiab] OR smart[tiab] OR mobile[tiab] OR app[tiab] OR apps[tiab] OR tablet*[tiab] OR touchscreen*[tiab] OR kiosk*[tiab] OR web-based[tiab] OR online[tiab] OR bedside[tiab] OR "information system*"[tiab] OR "ordering system*"[tiab] OR "monitoring system*"[tiab] OR "electronic health record*"[tiab] OR "electronic medical record*"[tiab] OR barcode*[tiab] OR RFID[tiab] OR sensor*[tiab] OR camera*[tiab] OR photograph*[tiab] OR imaging[tiab] OR algorithm*[tiab] OR dashboard*[tiab] OR "decision support"[tiab] OR "real time"[tiab] OR "real-time"[tiab])) OR (( "Food Loss and Waste"[Mesh] OR "Nutritional Status"[Mesh] OR "Nutrition Assessment"[Mesh] OR "Malnutrition"[Mesh] OR "Energy Intake"[Mesh] OR "food intake"[tiab] OR "dietary intake"[tiab] OR "nutritional intake"[tiab] OR "oral intake"[tiab] OR "energy intake"[tiab] OR "protein intake"[tiab] OR "nutrient intake"[tiab] OR "meal consumption"[tiab] OR "plate waste"[tiab] OR "plate wastage"[tiab] OR "food waste"[tiab] OR "meal waste"[tiab] OR "tray waste"[tiab] OR leftover*[tiab] OR "uneaten food"[tiab] OR "nutritional status"[tiab] OR "nutrition risk"[tiab] OR malnutrition[tiab]) AND ( "Artificial Intelligence"[Mesh] OR "Machine Learning"[Mesh] OR "Image Processing, Computer-Assisted"[Mesh] OR "artificial intelligence"[tiab] OR AI[tiab] OR "machine learning"[tiab] OR "deep learning"[tiab] OR "neural network*"[tiab] OR "computer vision"[tiab] OR "image recognition"[tiab] OR "food recognition"[tiab] OR "image analysis"[tiab] OR "image-based"[tiab] OR "image-assisted"[tiab] OR "digital photograph*"[tiab] OR camera*[tiab] OR sensor*[tiab] OR "smart tray*"[tiab] OR "automated dietary assessment"[tiab] OR "automatic dietary assessment"[tiab] OR "portion estimation"[tiab] OR "volume estimation"[tiab] OR "electronic scale*"[tiab] OR "weighing system*"[tiab] )) OR ( "electronic meal order*"[tiab] OR "electronic food order*"[tiab] OR "digital meal order*"[tiab] OR "electronic foodservice system*"[tiab] OR "electronic menu*"[tiab] OR "digital menu*"[tiab] OR "bedside meal order*"[tiab] OR "meal ordering system*"[tiab] OR "food ordering system*"[tiab] OR "patient-directed meal*"[tiab] OR "patient directed meal*"[tiab] OR "self-order*"[tiab] OR "self order*"[tiab] OR "self-service menu*"[tiab] OR "spoken menu*"[tiab] OR "room service"[tiab] OR "room-service"[tiab] OR "on-demand meal*"[tiab] OR "on demand meal*"[tiab] OR "a la carte"[tiab] OR " la carte"[tiab])) | 2,988 |
| Web of Science | 30 June 2026 | TS=((inpatient* OR hospital* OR hospitalized OR hospitalised OR ward* OR "acute care" OR "hospital patient*") AND ((( foodservice* OR "food service*" OR "dietary service*" OR "nutrition service*" OR "nutrition care" OR meal* OR menu* OR catering OR "hospital food*" OR "hospital diet*" OR "patient meal*" OR "meal tray*" OR "food tray*" OR "meal deliver*" OR "food deliver*" OR "meal order*" OR "food order*" OR "diet order*" OR "therapeutic diet*") AND ( digital* OR electronic* OR computer* OR technolog* OR informat* OR software OR automat* OR smart OR mobile OR app OR apps OR tablet* OR touchscreen* OR kiosk* OR web-based OR online OR bedside OR "information system*" OR "ordering system*" OR "monitoring system*" OR barcode* OR RFID OR sensor* OR camera* OR photograph* OR imaging OR algorithm* OR dashboard* OR "decision support" OR "real time" OR "real-time")) OR (( "food intake" OR "dietary intake" OR "nutritional intake" OR "oral intake" OR "energy intake" OR "protein intake" OR "meal consumption" OR "plate waste" OR "plate wastage" OR "food waste" OR "meal waste" OR "tray waste" OR leftover* OR "uneaten food" OR "nutritional status" OR "nutrition risk" OR malnutrition) AND ( "artificial intelligence" OR AI OR "machine learning" OR "deep learning" OR "neural network*" OR "computer vision" OR "image recognition" OR "food recognition" OR "image analysis" OR "image-based" OR "image assisted" OR "digital photograph*" OR camera* OR sensor* OR "smart tray*" OR "automated dietary assessment" OR "portion estimation" OR "volume estimation" OR "electronic scale*")) OR( "electronic meal order*" OR "electronic food order*" OR "digital meal order*" OR "electronic foodservice system*" OR "electronic menu*" OR "digital menu*" OR "bedside meal order*" OR "meal ordering system*" OR "food ordering system*" OR "patient-directed meal*" OR "self- order*" OR "self-service menu*" OR "spoken menu*" OR "room service" OR "on- demand meal*" OR "a la carte"))) | 2,435 |
| Scopus | 30 June 2026 | TITLE-ABS-KEY ( ( inpatient* OR hospitalized OR hospitalised OR "hospital patient*" OR "acute care" OR ward* ) AND ( foodservice* OR "food service*" OR "meal service*" OR "hospital meal*" OR "patient meal*" OR "meal order*" OR menu* OR catering OR tray* OR "room service" OR "dietary intake" OR "food intake" OR "nutritional intake" OR "meal consumption" OR "plate waste" OR "food waste" ) AND ( digital* OR electronic* OR computerized OR computerised OR technolog* OR " mobile application*" OR smartphone* OR tablet* OR touchscreen* OR "computer vision" OR "image-based" OR "image based" OR "automated dietary assessment" OR barcode* OR RFID OR "radio frequency identification" OR dashboard* OR algorithm* OR "decision support" OR "real- time monitoring" OR ( ( digital* OR electronic* OR bedside OR mobile OR online OR self ) W/3 ( meal* OR menu* OR order* OR foodservice* ) ) ) ) | 500 |
| Embase | 30 June 2026 | ( 'hospital patient'/exp OR 'hospitalization'/exp OR 'hospital'/exp OR inpatient*:ti,ab,kw OR hospitalized:ti,ab,kw OR hospitalised:ti,ab,kw OR 'hospital patient*':ti,ab,kw OR 'acute care':ti,ab,kw OR ward*:ti,ab,kw ) AND ( 'hospital food service'/exp OR 'dietary service'/exp OR foodservice*:ti,ab,kw OR 'food service*':ti,ab,kw OR 'meal service*':ti,ab,kw OR 'hospital meal*':ti,ab,kw OR 'patient meal*':ti,ab,kw OR 'meal order*':ti,ab,kw OR menu*:ti,ab,kw OR catering:ti,ab,kw OR tray*:ti,ab,kw OR 'room service':ti,ab,kw OR 'dietary intake':ti,ab,kw OR 'food intake':ti,ab,kw OR 'nutritional intake':ti,ab,kw OR 'meal consumption':ti,ab,kw OR 'plate waste':ti,ab,kw OR 'food waste':ti,ab,kw ) AND ( 'medical informatics'/exp OR 'hospital information system'/exp OR 'point of care system'/exp OR 'mobile application'/exp OR 'artificial intelligence'/exp OR 'image processing'/exp OR digital*:ti,ab,kw OR electronic*:ti,ab,kw OR computerized:ti,ab,kw OR computerised:ti,ab,kw OR tablet*:ti,ab,kw OR touchscreen*:ti,ab,kw OR smartphone*:ti,ab,kw OR 'mobile application*':ti,ab,kw OR 'computer vision':ti,ab,kw OR 'image based':ti,ab,kw OR 'automated dietary assessment':ti,ab,kw OR barcode*:ti,ab,kw OR rfid:ti,ab,kw OR 'radio frequency identification':ti,ab,kw OR dashboard*:ti,ab,kw OR algorithm*:ti,ab,kw OR 'decision support':ti,ab,kw OR ( (digital* OR electronic* OR bedside OR mobile OR online OR self) NEAR/3 (meal* OR menu* OR order* OR foodservice*) ):ti,ab,kw ) | 1,102 |

*Note.* Search syntax was adapted to the controlled vocabulary and field/proximity syntax of each database. Records retrieved denotes the database-level count returned by the final search before de-duplication. The table reproduces the complete strategies used for the four indexed bibliographic databases. IEEE Xplore, ACM Digital Library, CINAHL, trial registries, dissertation databases, and other grey-literature sources were not searched, and no separate backward or forward citation-chaining procedure was reported. This restriction may have reduced retrieval of engineering, nursing/allied-health, conference, or early-stage implementation evidence. Abbreviations: AI, artificial intelligence; EHR, electronic health record; /exp, exploded Emtree term; MeSH, Medical Subject Headings; MEDLINE, Medical Literature Analysis and Retrieval System Online; NEAR/3 and W/3, proximity operators within three words; RFID, radio-frequency identification; ti, title; ab, abstract; kw, author keyword; tiab, title/abstract; TITLE-ABS-KEY, title, abstract and keywords; TS, topic search.

**Supplementary Table S2. Available aggregate definitions and counts for full-text exclusions**

| **Primary exclusion category** | **Operational definition** | **Boundary rule** | **n** | **% of 715** |
| --- | --- | --- | --- | --- |
| Simulated leftovers; no real inpatient intake data | Laboratory, computational, synthetic, or nonclinical leftover data without a meal served to an inpatient, an actual returned tray, or a routine hospital foodservice record. | Controlled experiments using hospital food but no inpatient consumption were classified as staged meals rather than simulated leftovers. | 244 | 34.1% |
| Conference algorithm study; no clinical workflow validation | Full conference paper or proceeding describing an algorithm or device without testing in an operational inpatient foodservice or nutrition-care workflow using actual inpatient or returned-tray data. | Conference abstracts were ineligible as publication types; full proceedings were retained to full-text review only when sufficient methods were available. | 166 | 23.2% |
| Screening/diagnosis only; no eligible foodservice or intake function | Digital malnutrition screening, diagnostic, or risk-classification work not linked to meal ordering, diet orders, intake capture, foodservice delivery, or a nutrition-care response. | Reports with an implemented intake, meal, or foodservice function were assessed under the remaining eligibility criteria. | 148 | 20.7% |
| Digital attitudes only; no actual service use | Survey, interview, or qualitative report addressing opinions, intentions, or hypothetical acceptance without actual use of the digital system in an inpatient foodservice or nutrition-care service. | Usability or implementation studies were eligible only when participants interacted with an implemented or functioning system. | 93 | 13.0% |
| Staged hospital meals; no actual returned trays | Hospital foods were arranged, photographed, weighed, or otherwise tested under controlled conditions but were not consumed by inpatients and did not pass through routine returned-tray workflow. | Studies using actual returned trays or routine foodservice data remained eligible even when patient-level characteristics were unavailable. | 64 | 9.0% |
| **Total full-text reports excluded** |  |  | **715** | **100.0%** |

*Note.* The unit of exclusion was the report. Two reviewers independently assessed full texts; disagreements were resolved by discussion and, when required, consultation with a third reviewer. The retained review archive available for this revision contained only the aggregate category counts shown in this table. It did not contain a citation-level list of the 715 reports or the identifiers and retrieval-attempt history for the eight reports recorded as not retrieved. Those records therefore could not be reconstructed or added without risking misclassification. The counts and identifiers must be verified against the original screening platform or library export before resubmission; if that source cannot be recovered, this documentation gap should remain explicitly reported as a limitation. This table is not a citation-level exclusion list.

**Supplementary Table S3. Design-specific methodological considerations and role in the title-focused synthesis**

| **Report** | **Study design** | **Appraisal framework** | **Checklist items judged No/Unclear** | **Key methodological strengths** | **Main methodological concerns** | **Interpretation** | **Role in title-focused synthesis** |
| --- | --- | --- | --- | --- | --- | --- | --- |
| van Bakel et al., 2024 | Prospective repeated service evaluation with baseline and three annual follow-ups | JBI quasi-experimental checklist | Q2 U; Q3 N; Q4 N; Q5 N | Large number of meals, repeated annual follow-up and objective waste measurement. | No concurrent control; week-long snapshots; no patient-level intake/clinical outcomes. | Strong service/waste signal; limited clinical inference. | Direct outcome evidence: plate waste, meal frequency and cost |
| Svendsen et al., 2024 | Mixed-method development study: patient questionnaire plus two iterative think-aloud usability tests | MMAT 2018 mixed-methods criteria | 5.2 U; 5.3 U; 5.5 N | Patient preference survey plus iterative usability redesign; transparent SUS results. | Usability n=10; excluded vulnerable/very ill patients; no effectiveness evaluation. | Strong development evidence; exploratory implementation/generalizability. | Supporting context: ordering-system usability |
| Terp et al., 2024 | Mixed-method feasibility study with pre/post patient cohorts and qualitative evaluation | MMAT 2018 mixed-methods criteria | 5.5 N | Real-world pre/post intake measures and qualitative feasibility assessment. | Nonrandomized cohorts, single centre and possible concurrent changes. | Adequate feasibility/effect signal; some concern for attribution. | Direct outcome evidence: dietary intake and requirement attainment |
| Wray et al., 2026 | Mixed-method proof-of-concept service evaluation | MMAT 2018 mixed-methods criteria | 5.4 U; 5.5 N | Real-world family/staff data, safety validation process and multiple qualitative perspectives. | Short four-week pilot, small post sample, no direct intake/waste/clinical outcome and specialist setting. | Strong feasibility description; exploratory effectiveness evidence. | Supporting context: on-demand delivery feasibility |
| Ellick et al., 2025 | Implementation/quality-improvement study guided by Knowledge-to-Action and Donabedian frameworks | MMAT 2018 mixed-methods criteria | 5.2 U; 5.3 U; 5.4 U; 5.5 N | Framework-guided implementation; routine service, staff and safety data; transparent pathway description. | No control; descriptive variable denominators; multiple cointerventions and limited patient outcomes. | Strong implementation description; preliminary effectiveness evidence only. | Direct descriptive/supporting evidence: malnutrition care pathway |
| Maunder et al., 2022 | Two-stage validation and feasibility study | JBI diagnostic test accuracy checklist | Q1 U; Q4 U; Q5 U; Q7 U | Gold-standard WFR in Stage 1; routine-care feasibility in Stage 2; completeness and time quantified. | Short assessment, trained observers; Stage 2 used MI as reference and lacked patient outcomes. | Strong for measurement/workflow performance; not clinical effectiveness. | Measurement evidence: intake completeness and accuracy |
| Paulsen et al., 2020 | Parallel randomized controlled trial | JBI randomized controlled trial checklist | Q4 N; Q5 N; Q6 U | Random allocation; clinically relevant process and nutritional outcomes; low absolute attrition. | Participants/staff unblinded; single centre; modest sample and short exposure. | Most robust effectiveness evidence in the corpus, with some performance-bias concern. | Direct outcome evidence: nutritional status and nutrition care |
| Albaladejo et al., 2025 | Pilot criterion-validation study | JBI diagnostic test accuracy checklist | Q1 U; Q4 U; Q5 U; Q7 U | Real hospital trays, manual weighing reference, category-specific concordance. | Single-site pilot; image/component clustering; limited food categories and no patient outcomes. | Adequate technical pilot; external validation needed. | Measurement evidence: food-consumption estimation |
| Liu et al., 2026 | Comparative accuracy and usability study | JBI diagnostic test accuracy checklist; usability descriptive | Q1 U; Q4 U; Q5 U; Q7 U | Real returned trays, weighed/visual comparisons and formal SUS testing. | Liquid foods only, small nurse sample and repeated observations. | Adequate device pilot; accuracy requires further refinement. | Measurement evidence: automated intake estimation and usability |
| Ryu et al., 2024 | Prospective pilot validation study | JBI diagnostic test accuracy checklist | Q1 U; Q3 N; Q4 U; Q5 U; Q7 U; Q10 N | Prospective biological reference and clinically relevant sodium outcome. | Only 25/54 analyzed; small sample; diuretic and collection effects. | Exploratory proof of concept with substantial precision/attrition concerns. | Measurement evidence: sodium-intake estimation |
| Tagi et al., 2024 | Clinical development and criterion-validation study | JBI diagnostic test accuracy checklist | Q1 U; Q4 U; Q5 U; Q7 U | Real patient-served foods; weighed reference; comparison with two visual methods; nutrient metrics. | Single site, liquid foods only and repeated components. | Adequate clinical validation for liquid foods. | Measurement evidence: AI-assisted intake estimation |
| Munk et al., 2025 | One-day cross-sectional evaluation with historical 2019 comparison | JBI analytical cross-sectional checklist | Q5 U; Q6 N | Clinically relevant intake, malnutrition and downstream outcomes; explicit historical benchmark. | One-day cross-section, historical comparator, incomplete documentation and selected longer-stay population. | Useful contemporary gap/effect signal; not causal effectiveness evidence. | Direct outcome evidence: intake adequacy and nutritional status |
| Olufson et al., 2024 | Focused ethnography using observations and interviews | JBI qualitative checklist | Q1 U | Substantial ethnographic observation, multiple units and stakeholder perspectives. | Context-specific; no quantitative outcomes or technology-standardization. | Strong for explaining implementation and work-system mechanisms. | Supporting context: implementation and person-centred care |
| Barrington et al., 2018 | Comparative pre/post service evaluation | JBI quasi-experimental checklist | Q2 U; Q3 N; Q4 N; Q5 N | Objective ordered/consumed nutrient outcomes and clearly defined comparison periods. | Nonrandomized pre/post cohorts; unmeasured confounding; specialist oncology setting. | Adequate service-effect evidence with causal limitations. | Direct outcome evidence: dietary intake and plate waste |
| Barsha et al., 2023 | Retrospective repeated cross-sectional survey comparison | JBI analytical cross-sectional checklist | Q5 U; Q6 U | Multiple real-world foodservice models and adjusted analysis. | Nonrandomized model/site/time confounding; voluntary survey response; unequal group sizes. | Useful comparative satisfaction evidence; limited causal inference. | Supporting context: foodservice satisfaction |
| Jamison et al., 1996 | Comparative usability and cost evaluation; patients used both computerized and printed menus | JBI quasi-experimental checklist | Q5 N; Q6 U; Q9 U | Within-patient exposure to both formats; clear comparative outcomes and transparent cost assumptions. | Very small volunteer sample; no randomization/counterbalancing; self-reported short-term outcomes; dated technology. | Exploratory evidence; limited for causal or contemporary effectiveness inference. | Supporting context: menu acceptability and cost |
| Maunder et al., 2015 | Quasi-experimental pre/post comparison | JBI quasi-experimental checklist | Q2 U; Q3 N; Q4 N; Q5 N | Clearly defined pre/post systems; objective intake data and patient preference. | Nonrandomized cohorts, single site, potential secular/case-mix confounding. | Adequate for service-effect signal; some concern for causal inference. | Direct outcome evidence: dietary intake |
| McCray et al., 2018 | Retrospective pre/post routine-audit comparison | JBI quasi-experimental checklist | Q2 U; Q3 N; Q4 N; Q5 N | Multiple objective outcomes including intake, waste and cost; routine-care relevance. | Retrospective audit, nonconcurrent groups and possible secular changes. | Adequate real-world evidence; some concern for attribution. | Direct outcome evidence: dietary intake, plate waste and cost |
| Fisher et al., 2023 | Retrospective observational validation study | JBI analytical cross-sectional checklist | Q1 U; Q7 U | Large hospital-day dataset; agreement and multivariable association reported. | Retrospective single-site model, no external validation; predictive value attenuated after weight adjustment. | Exploratory predictive evidence requiring validation. | Direct/associational evidence: nutritional-risk identification |
| Paulsen et al., 2018 | Development and criterion-validation study | JBI diagnostic test accuracy checklist | Q1 U; Q4 U; Q5 U; Q7 U | Reference method using photography/partial weighing; individual-level agreement and usability assessed. | Small sample, two days, self-entry and no independent external validation. | Adequate early validation evidence. | Measurement evidence: dietary-intake recording |
| Roberts et al., 2017b | Qualitative interviews and focus groups | JBI qualitative checklist | Q1 U; Q6 U; Q7 U | Multiple professional groups; appropriate interviews/focus groups; implementation-focused analysis. | Single institution and perception-based outcomes. | Strong for local implementation determinants. | Supporting context: staff workflow and implementation |
| Roberts et al., 2017a | Qualitative usability study using think-aloud observation and semistructured interviews | JBI qualitative checklist | Q1 U; Q6 U; Q7 U | Appropriate think-aloud/interview methods; purposive multi-ward sample; transparent thematic findings. | Short exposure, voluntary sample and exclusion of patients unable to engage. | Strong for usability questions; transferability limited. | Supporting context: patient usability and equity |
| Ofei et al., 2015 | Prospective observational meal-level study | JBI analytical cross-sectional checklist | Q1 U; Q5 U; Q6 U | Prospective objective DIMS measurements; defined nutritional-risk assessment and statistical analysis. | Short single-site period; meal-level dependence and residual confounding. | Adequate observational evidence for associations. | Direct outcome evidence: plate waste |
| Ofei et al., 2019 | Pilot criterion-validation study | JBI diagnostic test accuracy checklist | Q1 U; Q4 U; Q5 U; Q7 U | Reference comparison and inter-rater reliability reported. | Only 17 meals; trained assessors and narrow setting. | Exploratory pilot; low precision/generalizability. | Measurement evidence: intake and plate-waste estimation |
| Rattray et al., 2018 | Prospective observational meal-process audit | JBI analytical cross-sectional checklist | Q1 U; Q5 U; Q6 U | Large meal sample; explicit therapeutic-diet error framework and severity assessment. | No nondigital comparator; one hospital; observer-dependent error classification. | Adequate for mapping safety failures, not intervention effect. | Supporting context: diet safety and workflow |

*Note.* Appraisal was used to contextualize confidence, transferability, and applicability rather than to calculate a universal cross-design score or exclude studies automatically. The exact design-specific JBI checklist or MMAT 2018 mixed-methods criteria are named for each report. The item-level column reproduces applicable criteria judged No (N) or Unclear (U); Yes and Not Applicable judgments are omitted for readability, and criterion numbering follows the named tool. Criterion-validation studies used the JBI diagnostic test accuracy checklist as the closest design-specific tool, with nonapplicable diagnostic items treated as Not Applicable. Abbreviations: DIMS, Dietary Intake Monitoring System; FRC, food-record chart; JBI, Joanna Briggs Institute; MI, Mobile Intake; MMAT, Mixed Methods Appraisal Tool; MST, Malnutrition Screening Tool; N, No; NR, not reported; RCT, randomized controlled trial; SUS, System Usability Scale; U, Unclear; WFR, weighed food record.

**Supplementary Table S4A. Detailed bibliographic, setting and population characteristics with evidence role**

| **Report** | **Full title and journal** | **Country and hospital setting** | **Design** | **Population/data source** | **Sample** | **Age/sex information** | **Digital technology** | **Role in title-focused synthesis** | **Identifiers** |
| --- | --- | --- | --- | --- | --- | --- | --- | --- | --- |
| van Bakel et al., 2024 | Personalization in mitigating food waste and costs in hospitalization. Clinical Nutrition. | Netherlands; Eight wards on two hospital floors | Prospective repeated service evaluation with baseline and three annual follow-ups | Meals served to hospitalized patients | Traditional system 4,361 meals; digital system 7,815 meals (12,176 total) | Not applicable (meal-level service evaluation) | Patient-centred digital three-channel foodservice: bedside point-of-service ordering, telephone call-and-order and bistro access | Direct outcome evidence: plate waste, meal frequency and cost | DOI: 10.1016/j.clnu.2024.08.005; PMID: 39167983 |
| Svendsen et al., 2024 | Development of an electronic food ordering system and a la carte menu: enhancing patient involvement in nutritional care. Clinical Nutrition ESPEN. | Denmark; Medical and oncology wards, Herlev-Gentofte Hospital | Mixed-method development study: patient questionnaire plus two iterative think-aloud usability tests | Hospitalized patients | Questionnaire n=99; usability test 1 n=6; usability test 2 n=4 | Questionnaire: median 65.5 years (IQR 52-76), 51.5% female; usability samples aged 11-81 and 31-70 years | Tablet-based patient-centred electronic food-ordering system with à la carte menu and meal diary | Supporting context: ordering-system usability | DOI: 10.1016/j.clnesp.2024.01.014; PMID: 38479944 |
| Terp et al., 2024 | An educative nutritional intervention supporting older hospital patients to eat sufficiently using eHealth: a mixed methods feasibility and pilot study. BMC Geriatrics. | Denmark; Internal medicine hospital wards | Mixed-method feasibility study with pre/post patient cohorts and qualitative evaluation | Hospitalized adults aged ≥65 years | Pre-intervention n=65; post-intervention n=65 | Pre-intervention: 82±7.2 years, 44% female; post-intervention: 83±5.8 years, 57% female | Food'n'Go eHealth system for food ordering, intake registration, individualized feedback and nutrition education | Direct outcome evidence: dietary intake and requirement attainment | DOI: 10.1186/s12877-023-04582-x; PMID: 38177992 |
| Wray et al., 2026 | Food on demand delivery service: from hospitality to hospital-trialling a proof of concept in a specialist children's hospital. BMJ Open Quality. | United Kingdom; Six long-stay wards in a specialist children's hospital | Mixed-method proof-of-concept service evaluation | Hospitalized children/families, ward staff and catering staff | Baseline survey 101 families; proof-of-concept survey 26 families; interviews 18 parents and 11 ward staff; focus groups 4 ward and 4 catering staff | Pediatric setting (hospital serves ages 0-18 years); detailed participant ages NR | Web-based food-on-demand ordering application with ward validation and kitchen delivery | Supporting context: on-demand delivery feasibility | DOI: 10.1136/bmjoq-2025-003465; PMID: 41571399 |
| Ellick et al., 2025 | Harnessing delegation and technology to identify and manage malnutrition in a digital hospital: an implementation study. Nutrition & Dietetics. | Australia; New metropolitan digital public hospital with surgical, geriatric and rehabilitation inpatient services, Queensland | Implementation/quality-improvement study guided by Knowledge-to-Action and Donabedian frameworks | Inpatient dietetics service, dietetic assistants and hospital-wide audit population | Service-level implementation data; staff survey and hospital-wide one-day malnutrition audit; exact patient/service denominators vary by measure | Hospital-wide audit patients: median 69 years (IQR 58-79); sex NR | Electronic screening and meal-intake tracking with automated dietetic referrals and delegated care pathways | Direct descriptive/supporting evidence: malnutrition care pathway | DOI: 10.1111/1747-0080.12913; PMID: 39551986 |
| Maunder et al., 2022 | Validation of an electronic food intake tool and its usability and efficacy in the healthcare setting. Journal of Human Nutrition and Dietetics. | Australia; Two major tertiary referral hospitals; usual-care rehabilitation ward for Stage 2 | Two-stage validation and feasibility study | Hospital meal trays and rehabilitation inpatients | Stage 1: 90 meal trays; Stage 2: 45 individuals and 210 meal trays | Stage 1 not applicable (meal trays); Stage 2 participant age/sex NR | Mobile Intake (MI), a touch-screen electronic food-intake and plate-waste tool integrated with nutrient data | Measurement evidence: intake completeness and accuracy | DOI: 10.1111/jhn.12969; PMID: 34846076 |
| Paulsen et al., 2020 | Effects of using the MyFood decision support system on hospitalized patients' nutritional status and treatment: a randomized controlled trial. Clinical Nutrition. | Norway; Oslo University Hospital | Parallel randomized controlled trial | Hospitalized adults at nutritional risk | n=100 randomized: intervention n=49; control n=51 | Mean 51.9±14.0 years; male 71% in the intervention group and 49% in the control group | MyFood patient tablet app plus web-based decision support for nurses/clinicians [Linked cluster: MyFood development and randomized evaluation.] | Direct outcome evidence: nutritional status and nutrition care | DOI: 10.1016/j.clnu.2020.03.012; PMID: 32241711 |
| Albaladejo et al., 2025 | Assessing real-life food consumption in hospital with an automatic image recognition device: a pilot study. Clinical Nutrition ESPEN. | France; Hospital ward meal service | Pilot criterion-validation study | Actual inpatient meal trays, including pre-consumption test trays and post-meal returned trays | Annotation: 246 food components; testing: 368 components from 183 images | Not applicable (image/component-level validation) | Locally processed RGB-D automatic image-recognition prototype | Measurement evidence: food-consumption estimation | DOI: 10.1016/j.clnesp.2025.05.017; PMID: 40389093 |
| Liu et al., 2026 | Usability and accuracy of an artificial intelligence-based automatic meal tray photography device for estimating liquid food intake in healthcare settings. Clinical Nutrition ESPEN. | Japan; Hospital wards serving liquid diets | Comparative accuracy and usability study | Actual post-consumption hospital liquid-food items and nursing users | 174 food items over 51 days; 18 nurses; usability observations: automatic device 144 items, manual tablet 118 items | Not applicable to food-item dataset; nurse demographics NR | AI-based automatic meal-tray photography device compared with manual tablet photography | Measurement evidence: automated intake estimation and usability | DOI: 10.1016/j.clnesp.2025.11.131; PMID: 41260254 |
| Ryu et al., 2024 | Sodium Intake Estimation in Hospital Patients Using AI-Based Imaging: Prospective Pilot Study. JMIR Formative Research. | South Korea; Single hospital inpatient setting | Prospective pilot validation study | Hospitalized adults with complete meal images and 24-h urinary sodium | 54 enrolled; 25 completed analysis | Median 64 years (IQR 53-74); 68% male | AI-based before/after meal imaging for sodium-intake estimation | Measurement evidence: sodium-intake estimation | DOI: 10.2196/48690; PMID: 38363594 |
| Tagi et al., 2024 | A Food Intake Estimation System Using an Artificial Intelligence-Based Model for Estimating Leftover Hospital Liquid Food in Clinical Environments: Development and Validation Study. JMIR Formative Research. | Japan; Two wards, Tokushima University Hospital | Clinical development and criterion-validation study | Actual liquid-food dishes served to hospitalized patients | 100 patient meals; 300 dishes/components | Not applicable (meal/dish-level validation; patient demographics not reported) | Mobile image upload with AI model estimating leftover liquid food and nutrient intake | Measurement evidence: AI-assisted intake estimation | DOI: 10.2196/55218; PMID: 39500491 |
| Munk et al., 2025 | The effect of a digital energy- and protein-dense food concept with a nutritional tracker on dietary intake in hospitalized patients at nutritional risk: a one-day cross-sectional study. Clinical Nutrition ESPEN. | Denmark; Copenhagen University Hospital-Herlev and Gentofte; adult wards excluding intensive, palliative, emergency and maternal care | One-day cross-sectional evaluation with historical 2019 comparison | Adults hospitalized >4 days | n=124 overall; dietary intake assessed in n=69; 81 (65%) at nutritional risk | Median 75 years (IQR 66-83); 52% female | "Digital Delights of Herlev Hospital": tablet-based self-ordering, energy-/protein-dense à la carte food and nutritional tracker | Direct outcome evidence: intake adequacy and nutritional status | DOI: 10.1016/j.clnesp.2025.05.037; PMID: 40447222 |
| Olufson et al., 2024 | Enhancing or impeding? The influence of digital systems on interprofessional practice and person-centred care in nutrition care systems across rehabilitation units. Nutrition & Dietetics. | Australia; Three subacute rehabilitation units | Focused ethnography using observations and interviews | Patients and staff involved in inpatient nutrition care | 58 h fieldwork; 125 people observed and 77 interviewed (165 unique participants) | NR for the combined patient, support-person and staff sample | Multiple digital systems supporting electronic documentation, foodservice and nutrition-care communication | Supporting context: implementation and person-centred care | DOI: 10.1111/1747-0080.12846; PMID: 37850243 |
| Barrington et al., 2018 | Engaging the patient: improving dietary intake and meal experience through bedside terminal meal ordering for oncology patients. Journal of Human Nutrition and Dietetics. | Australia; Specialist oncology hospital | Comparative pre/post service evaluation | Hospitalized oncology patients | Paper menu n=96; bedside meal-ordering system n=105 (total n=201) | Paper menu: mean 60.5 years (range 19-93), 54% male; bedside system: mean 65.0 years (range 18-88), 53% male | Bedside terminal meal-ordering system | Direct outcome evidence: dietary intake and plate waste | DOI: 10.1111/jhn.12573; PMID: 29963727 |
| Barsha et al., 2023 | Is meal order timing and flexibility key to improving patient foodservice satisfaction?. Journal of Human Nutrition and Dietetics. | Australia; Hospital foodservice services using four ordering models | Retrospective repeated cross-sectional survey comparison | Hospital inpatients responding to foodservice satisfaction surveys | n=405: traditional menu 63; call-at-point-of-service 44; BMOS 149; room service 149 | Mean age 47.6-58.5 years across ordering models; 76.7%-91.1% female | Digital/technology-supported ordering models with differing timing and flexibility | Supporting context: foodservice satisfaction | DOI: 10.1111/jhn.13186; PMID: 37335683 |
| Jamison et al., 1996 | A computerized interactive menu selector system for hospitals. Journal of the American Dietetic Association. | United States; One hospital wing in a large metropolitan hospital, north-central Texas | Comparative usability and cost evaluation; patients used both computerized and printed menus | Hospital patients/volunteers aged 7-78 years | 50 recruited; 47 questionnaire respondents analyzed | 7-78 years; sex distribution not reported | Computerized interactive menu selector accessed through the hospital in-house video/electronic bulletin-board system | Supporting context: menu acceptability and cost | DOI: 10.1016/S0002-8223(96)00277-5; PMID: 8841169 |
| Maunder et al., 2015 | Energy and protein intake increases with an electronic bedside spoken meal ordering system compared to a paper menu in hospital patients. Clinical Nutrition ESPEN. | Australia; Five wards in a 210-bed tertiary private hospital, Sydney | Quasi-experimental pre/post comparison | Adult inpatients receiving oral diets | Paper menu n=54; electronic bedside system n=65 (total n=119) | Paper menu: 65±14 years, 69% female; electronic system: 66±13 years, 59% female | Electronic bedside spoken meal-ordering system | Direct outcome evidence: dietary intake | DOI: 10.1016/j.clnesp.2015.05.004; PMID: 28531390 |
| McCray et al., 2018 | Bedside Menu Ordering System increases energy and protein intake while decreasing plate waste and food costs in hospital patients. Clinical Nutrition ESPEN. | Australia; 126-bed public acute hospital; medical, surgical and oncology services, Brisbane | Retrospective pre/post routine-audit comparison | Adult hospital patients receiving oral diets | Traditional menu n=84; BMOS n=104 (total n=188) | Traditional menu: 63±19 years, 57% female; BMOS: 72±15 years, 56% female | Bedside Menu Ordering System (BMOS) | Direct outcome evidence: dietary intake, plate waste and cost | DOI: 10.1016/j.clnesp.2018.04.012; PMID: 29908685 |
| Fisher et al., 2023 | Using an interactive nutrition technology platform to predict malnutrition risk. Journal of Human Nutrition and Dietetics. | Australia; 99-bed hospital | Retrospective observational validation study | Adult inpatients with electronic meal/intake and screening data | 216 patients; 1,783 hospital-days; inter-rater dataset 912 food items | Median 74 years (IQR 23; range 18-104); 56.9% male | Interactive Nutrition Dashboard integrating food provision, intake records and risk information | Direct/associational evidence: nutritional-risk identification | DOI: 10.1111/jhn.13088; PMID: 36083834 |
| Paulsen et al., 2018 | A Dietary Assessment App for Hospitalized Patients at Nutritional Risk: Development and Evaluation of the MyFood App. JMIR mHealth and uHealth. | Norway; Gastrointestinal surgery and hematology departments, Oslo University Hospital | Development and criterion-validation study | Hospitalized patients at nutritional risk | n=32 (gastrointestinal surgery n=18; hematology n=14) | Mean 51 years; range 17-77; 69% male | MyFood tablet application with patient-information, dietary-assessment and intake-versus-requirement modules [Linked cluster: MyFood development and randomized evaluation.] | Measurement evidence: dietary-intake recording | DOI: 10.2196/mhealth.9953; PMID: 30194059 |
| Roberts et al., 2017b | Hospital staffs' perceptions of an electronic program to engage patients in nutrition care at the bedside: a qualitative study. BMC Medical Informatics and Decision Making. | Australia; Same five wards in a tertiary teaching hospital | Qualitative interviews and focus groups | Hospital staff involved in nutrition and foodservice | n=19: 10 nurses, 3 dietitians, 2 nutrition assistants, 2 foodservice staff and 2 doctors | Age NR; 89% female | Bedside electronic program supporting patient engagement in nutrition care [Linked cluster: same bedside electronic foodservice program as Roberts et al., 2017a.] | Supporting context: staff workflow and implementation | DOI: 10.1186/s12911-017-0495-4; PMID: 28693472 |
| Roberts et al., 2017a | Technology to engage hospitalised patients in their nutrition care: a qualitative study of usability and patient perceptions of an electronic foodservice system. Journal of Human Nutrition and Dietetics. | Australia; Five wards (oncology, renal, orthopaedic, respiratory and vascular) in a tertiary teaching hospital | Qualitative usability study using think-aloud observation and semistructured interviews | Adult hospitalized patients | n=32 | Mean 54.4±16.6 years; range 22-80; 50% female | Bedside electronic foodservice and nutrition-care system [Linked cluster: same bedside electronic foodservice program as Roberts et al., 2017b.] | Supporting context: patient usability and equity | DOI: 10.1111/jhn.12467; PMID: 28211190 |
| Ofei et al., 2015 | Effect of meal portion size choice on plate waste generation among patients with different nutritional status: an investigation using Dietary Intake Monitoring System. Appetite. | Denmark; Two wards, Aalborg University Hospital | Prospective observational meal-level study | Hospitalized patients with differing nutritional-risk status | 71 patients; 256 meals (142 lunch, 114 supper) | Mean 65.6±13.5 years; female 50% among patients not at nutritional risk and 55% among those at risk | Dietary Intake Monitoring System (DIMS) combining pre/post images, integrated weighing and RFID identification [Linked platform family: DIMS, site/version-specific evaluation.] | Direct outcome evidence: plate waste | DOI: 10.1016/j.appet.2015.04.043; PMID: 25881858 |
| Ofei et al., 2019 | Validation of a novel image-weighed technique for monitoring food intake and estimation of portion size in hospital settings: a pilot study. Public Health Nutrition. | Denmark; Hospital meal-service setting, Odense, using DIMS 2.0 | Pilot criterion-validation study | Actual hospital patient meals | 17 patient meals | Not applicable (meal-level validation; patient demographics not reported) | Image-weighed DIMS 2.0 technique [Linked platform family: DIMS 2.0, site/version-specific evaluation.] | Measurement evidence: intake and plate-waste estimation | DOI: 10.1017/S1368980018001064; PMID: 29759093 |
| Rattray et al., 2018 | Identifying errors in meals provided to and sourced by patients on therapeutic diets in hospital. Asia Pacific Journal of Clinical Nutrition. | Australia; Six medical and surgical wards in a tertiary teaching hospital | Prospective observational meal-process audit | Adult inpatients prescribed one of 12 therapeutic diets | 167 patients; 906 meals | Mean 55.4±17.5 years; 57% female | Electronic foodservice system linking diet orders, menu planning, tray assembly and delivery | Supporting context: diet safety and workflow | DOI: 10.6133/apjcn.072017.10; PMID: 29737799 |

*Note.* Each row represents one included report, not necessarily one unique technology or implementation. Explicit linked clusters were: MyFood (Paulsen et al., 2018 development study and Paulsen et al., 2020 randomized evaluation); the Roberts bedside electronic foodservice program (paired 2017 patient- and staff-focused reports from the same hospital implementation); and the DIMS platform family (Ofei et al., 2015 and 2019, treated as site/version-specific evaluations). These reports were linked during interpretation and not treated as independent replication of a technology. Because some reports did not name products or institutions, an exact unique-system or hospital count could not be determined without assumptions. Sample size is presented using the source unit, including patients, meals, trays, components, hospital-days, respondents, or observations. Age and sex are reported only when applicable and available. Abbreviations: AI, artificial intelligence; BMOS, Bedside Menu Ordering System; DIMS, Dietary Intake Monitoring System; DOI, digital object identifier; IQR, interquartile range; JMIR, Journal of Medical Internet Research; N/A, not applicable; NR, not reported; PMID, PubMed identifier; RGB-D, red-green-blue plus depth imaging; RFID, radio-frequency identification; SD, standard deviation.

**Supplementary Table S4B. Detailed digital-technology characteristics and linkage to title outcomes**

| **Report** | **Technology/system** | **Device/interface** | **Primary users** | **Data input** | **Digital/automated output** | **Service-chain stage** | **Comparator/reference** | **System integration and human oversight** | **Title-outcome linkage** |
| --- | --- | --- | --- | --- | --- | --- | --- | --- | --- |
| van Bakel et al., 2024 | Patient-centred digital three-channel foodservice: bedside point-of-service ordering, telephone call-and-order and bistro access | Tablet/bedside terminal | Patients, nutritional assistants, dietitians, kitchen and ward staff | Digitally registered patient choices, patient ID/diet restrictions and waste weights | Live order history, diet-rule display and foodservice demand information | Ordering, diet restriction verification, decentralized production/delivery, meal tracking and waste collection | Traditional paper-based, preordered, preplated system | Patient identification and diet restrictions available digitally at point of service; Human oversight: Nutritional assistants and dietitians verified choices/restrictions | Plate waste; meal frequency; economic outcome |
| Svendsen et al., 2024 | Tablet-based patient-centred electronic food-ordering system with à la carte menu and meal diary | Tablet/bedside terminal | Patients; staff assistance anticipated for some patients | Food preferences, meal orders and optional intake-diary entries | Electronic ordering, scheduling and display of meal diary/information | Menu design, self-ordering, order modification and meal-intake diary | Usability before vs after interface refinement; no clinical comparator | Hospital foodservice concept; clinical-system integration not evaluated; Human oversight: Researchers observed think-aloud testing; clinical staff supported recruitment | Supporting usability evidence |
| Terp et al., 2024 | Food'n'Go eHealth system for food ordering, intake registration, individualized feedback and nutrition education | Digital platform/system; device details as reported | Older patients and ward staff | Food orders, actual intake and patient information | Calculates energy/protein intake relative to needs and displays feedback/education | Meal ordering, intake monitoring, requirement feedback and patient education | Pre-implementation usual care | Hospital foodservice and nutrition-care workflow; detailed EHR interface NR; Human oversight: Staff supported patients and could review data | Dietary intake and requirement attainment |
| Wray et al., 2026 | Web-based food-on-demand ordering application with ward validation and kitchen delivery | Digital platform/system; device details as reported | Families/patients, ward staff and catering staff | Web orders and ward diet/allergy validation | Electronic order transmission and tracking; clinical validation remained manual | Family order, ward validation for allergies/diet, kitchen preparation and targeted delivery within 25 min | Baseline foodservice experience before proof of concept | Ward-kitchen workflow; broader clinical-system linkage NR; Human oversight: Ward staff validated all orders before kitchen action | Supporting delivery evidence |
| Ellick et al., 2025 | Electronic screening and meal-intake tracking with automated dietetic referrals and delegated care pathways | Digital platform/system; device details as reported | Nurses, dietetic assistants and dietitians | MST scores, meal/snack intake and clinical nutrition assessment data | Automatic referral generation, real-time intake reports and task routing | MST screening, routine meal/snack intake tracking, automated referral, dietetic-assistant action and escalation to dietitian | No concurrent control; implementation benchmarks and audit measures | Electronic medical record and digital hospital food/nutrition systems; Human oversight: Protocolized dietetic-assistant tasks with dietitian escalation and review | Nutritional status and nutrition-care implementation |
| Maunder et al., 2022 | Mobile Intake (MI), a touch-screen electronic food-intake and plate-waste tool integrated with nutrient data | Digital platform/system; device details as reported | Foodservice staff/student dietitians; output for clinicians | Served menu items and percentage consumed (0%, 25%, 50%, 75%, 100%) | Automatic macro-/micronutrient analysis and electronic storage/reporting | Tray collection, visual percentage selection, automated nutrient analysis and real-time access | Weighed food record (WFR) and paper food-record charts (FRC) | CBORD foodservice/nutrient database; clinical data available in real time; Human oversight: Trained student dietitians and accredited practicing dietitians | Measurement validity and data completeness |
| Paulsen et al., 2020 | MyFood patient tablet app plus web-based decision support for nurses/clinicians [Linked cluster: MyFood development and randomized evaluation.] | Tablet/bedside terminal | Patients, nurses and clinical nutrition staff | Patient characteristics and food/fluid intake | Calculates requirements/intake, flags deficits and generates tailored nutrition recommendations/care-plan content | Daily intake recording, needs comparison, individualized recommendations and care-plan support | Usual nutritional care | Web clinical interface; documentation support; full EHR integration NR; Human oversight: Nurses and clinicians reviewed and implemented recommendations | Nutritional status and nutrition care |
| Albaladejo et al., 2025 | Locally processed RGB-D automatic image-recognition prototype | Camera/imaging device | Foodservice/clinical nutrition personnel | RGB and depth images of main-course components | Food segmentation and quantity estimation performed locally without cloud processing | Automatic tray detection, RGB-D imaging, segmentation, volume/weight estimation and consumption calculation | Manual weighing of meal components | Prototype designed for hospital tray workflow; not yet direct patient-care integration; Human oversight: Manual component weighing and expert annotation | Measurement validity: food consumption |
| Liu et al., 2026 | AI-based automatic meal-tray photography device compared with manual tablet photography | Fixed automatic RGB-D camera device; manual tablet-photography comparator | Nurses/ward and nutrition staff | Tray photographs of liquid foods | Automatic image capture and AI remaining-amount/intake estimation | Automatic tray photography at collection and AI intake estimation | Weighed food method and nurse visual estimation/manual photography | Placed in healthcare meal-tray workflow; routine EHR linkage NR; Human oversight: Nurses and weighed reference measurements | Measurement validity: intake and leftovers |
| Ryu et al., 2024 | AI-based before/after meal imaging for sodium-intake estimation | Before/after meal camera-imaging workflow with AI analysis | Patients/research staff; intended for clinicians | Pre- and post-meal images and menu nutrient information | AI food/portion analysis and daily sodium-intake estimate | Meal imaging, consumed-portion estimation and sodium calculation | 24-h urinary sodium excretion | Research workflow; routine EHR integration NR; Human oversight: Clinical/research review; urinary collection as reference | Measurement validity: sodium intake |
| Tagi et al., 2024 | Mobile image upload with AI model estimating leftover liquid food and nutrient intake | Camera/imaging device | Nurses, dietitians and other clinical staff | Photographs of post-meal liquid foods | AI leftover percentage and energy/protein/fat/carbohydrate estimation | Post-meal photography, leftover estimation and nutrient-intake calculation | Weighed reference; dietitian image-based visual estimate; nurse direct visual estimate | Clinical mobile workflow; wider EHR integration NR; Human oversight: Dietitian/nurse comparisons and weighing reference | Measurement validity: dietary intake |
| Munk et al., 2025 | "Digital Delights of Herlev Hospital": tablet-based self-ordering, energy-/protein-dense à la carte food and nutritional tracker | Tablet/bedside terminal | Patients and ward/foodservice staff | Patient orders, registered intake, screening and clinical outcomes | Digital ordering and tracker comparing intake with needs | Tablet meal ordering, intake registration, nutrition-risk screening and requirement monitoring | Historical 2019 hospital assessment | Whole-hospital digital foodservice; exact EHR linkage NR; Human oversight: Clinical staff completed screening/assessment and verified intake | Dietary intake; nutritional status; downstream outcomes |
| Olufson et al., 2024 | Multiple digital systems supporting electronic documentation, foodservice and nutrition-care communication | Digital platform/system; device details as reported | Patients, nurses, dietitians, assistants, medical and foodservice staff | Routine digital documentation and orders; qualitative observations/interviews | System-specific; electronic visibility and task routing, with variable interoperability | Documentation, ordering, communication, interdisciplinary practice and person-centred nutrition care | Cross-unit contextual comparison; no formal intervention comparator | Multiple systems with both enabling and siloing effects; Human oversight: Professional review and local workflows | Supporting implementation evidence |
| Barrington et al., 2018 | Bedside terminal meal-ordering system | Tablet/bedside terminal | Patients and foodservice/nutrition staff | Bedside electronic meal orders | Electronic menu display and order capture | Patient meal selection and electronic transmission of orders | Paper menu system | Hospital foodservice order production; EHR integration NR; Human oversight: Staff assistance; approximately 60% accessed independently | Dietary intake; plate waste |
| Barsha et al., 2023 | Digital/technology-supported ordering models with differing timing and flexibility | Digital platform/system; device details as reported | Patients, nutrition assistants and foodservice staff | Patient meal orders and satisfaction surveys | Varied by model; electronic order capture in BMOS/room-service pathways | Meal-order timing, order method and foodservice delivery | Traditional menu, call-at-point-of-service, bedside electronic ordering and room service | Hospital foodservice systems; detailed EHR linkage NR; Human oversight: Foodservice/nutrition staff supported ordering | Supporting service evidence |
| Jamison et al., 1996 | Computerized interactive menu selector accessed through the hospital in-house video/electronic bulletin-board system | Bedside television interface with touch-tone telephone keypad, linked to a central computer and printer | Patients; hospital foodservice personnel | Patient navigation and menu selections | Electronic presentation and submission of menu selections; monthly cost model | Menu display and patient meal selection | Printed paper menu | Hospital in-house video/electronic bulletin-board network; downstream integration NR; Human oversight: Foodservice staff supported implementation | Supporting service evidence |
| Maunder et al., 2015 | Electronic bedside spoken meal-ordering system | Tablet/bedside terminal | Patients and nutrition assistants | Verbal patient choices entered electronically at bedside | Electronic order capture and transmission; nutrient intake calculated from served/consumed items | Bedside meal ordering and direct patient-nutrition-assistant interaction | Traditional paper menu | Foodservice ordering workflow; EHR integration NR; Human oversight: Nutrition assistants assisted all orders | Dietary intake |
| McCray et al., 2018 | Bedside Menu Ordering System (BMOS) | Tablet/bedside terminal | Patients and nutrition assistants | Electronic patient meal orders and observed plate waste | Real-time order capture, menu availability and nutrient calculations | Bedside order taking, order transmission, production and tray-return waste assessment | Traditional menu system | Hospital foodservice platform; EHR integration NR; Human oversight: Nutrition assistants entered and verified orders | Dietary intake; plate waste; economic outcome |
| Fisher et al., 2023 | Interactive Nutrition Dashboard integrating food provision, intake records and risk information | Digital platform/system; device details as reported | Nurses, dietitians and clinical teams | Ordered/provided food, estimated intake, MST and weight | Dashboard risk categorization and visualization of longitudinal intake | Meal data capture, intake visualization and risk stratification | Malnutrition Screening Tool (MST), with additional analysis including body weight | Hospital electronic foodservice/clinical data platform; Human oversight: Clinical nutrition staff interpreted dashboard outputs | Nutritional-risk identification |
| Paulsen et al., 2018 | MyFood tablet application with patient-information, dietary-assessment and intake-versus-requirement modules [Linked cluster: MyFood development and randomized evaluation.] | Tablet/bedside terminal | Patients; nurses/health professionals enter clinical data | Patient characteristics and selected/consumed food and beverages | Calculates energy, protein and fluid intake and compares intake with estimated needs | Patient intake recording, nutrient calculation and comparison with individual requirements | Digital meal photography combined with partial weighing | Standalone prototype for clinical use; later versions linked to web decision support; Human oversight: Nurse/health professional entered clinical information; research reference assessment | Measurement validity: dietary intake |
| Roberts et al., 2017b | Bedside electronic program supporting patient engagement in nutrition care [Linked cluster: same bedside electronic foodservice program as Roberts et al., 2017a.] | Tablet/bedside terminal | Nurses, dietitians, doctors, nutrition assistants and foodservice personnel | Staff interaction with program and qualitative implementation feedback | Electronic access to foodservice/nutrition functions; exact algorithms NR | Patient engagement, foodservice use, nutrition monitoring and professional communication | None | Designed for bedside nutrition-care workflow; interoperability limitations discussed; Human oversight: Professional review and role-based use | Supporting implementation evidence |
| Roberts et al., 2017a | Bedside electronic foodservice and nutrition-care system [Linked cluster: same bedside electronic foodservice program as Roberts et al., 2017b.] | Tablet/bedside terminal | Patients, with staff assistance when required | Patient interactions, menu/foodservice selections and qualitative feedback | Electronic display and capture of nutrition/foodservice information; exact clinical algorithms NR | Electronic foodservice navigation, meal-related information and patient participation in nutrition care | None | Hospital foodservice system; degree of EHR linkage NR; Human oversight: Researcher observed think-aloud use; staff assistance available | Supporting implementation evidence |
| Ofei et al., 2015 | Dietary Intake Monitoring System (DIMS) combining pre/post images, integrated weighing and RFID identification [Linked platform family: DIMS, site/version-specific evaluation.] | Camera/imaging device | Foodservice/research staff; outputs intended for clinical nutrition monitoring | Meal identity, weights and images before and after consumption | Links tray identity with image and weight data to calculate consumption and waste | Meal production/identification, pre/post weighing, intake and plate-waste measurement | No intervention comparator; analyses by portion size, mealtime and nutritional-risk status | Hospital meal-service workflow; EHR integration NR; Human oversight: Research/foodservice personnel verified measurements | Plate waste |
| Ofei et al., 2019 | Image-weighed DIMS 2.0 technique [Linked platform family: DIMS 2.0, site/version-specific evaluation.] | Camera/imaging device | Trained assessors/foodservice or nutrition personnel | Meal images and weights | Image-weight linkage and nutrient estimation | Before/after meal imaging and weighing for portion and intake estimation | Manual weighing/reference assessment | Hospital tray workflow; EHR integration NR; Human oversight: Trained assessors and reference measurements | Measurement validity: intake and plate waste |
| Rattray et al., 2018 | Electronic foodservice system linking diet orders, menu planning, tray assembly and delivery | Digital platform/system; device details as reported | Dietitians, foodservice staff, nurses and patients | Electronic diet orders and meal-order/production data | Diet-rule application and electronic meal production information | Therapeutic diet order, menu planning, tray assembly, ward delivery and consumption | No intervention comparator; error location classified across workflow stages | Foodservice system linked to therapeutic-diet workflow; exact EHR interface NR; Human oversight: Dietitian/researcher audited actual meals and errors | Supporting safety evidence |

*Note.* Technology functions are described at the level reported in each source. The four review categories were operationalized as patient-facing ordering/on-demand tools; intake monitoring, dashboards, and decision support; image-, weight-, sensor-, RFID-, or AI-assisted measurement; and integrated foodservice, diet-safety, and workflow systems. Multicomponent systems were assigned to every applicable category, while the primary evidence role followed the function most directly linked to the reported outcome. NR means that integration or interoperability was not reported and does not demonstrate absence. Human oversight denotes reported staff review, diet/allergy validation, data verification, or clinical escalation. The comparator/reference may be concurrent, historical, criterion-based, or absent according to design. Abbreviations: AI, artificial intelligence; AI-automatic, photographs captured by the automatic device and analyzed by the AI model; AI-manual, tablet-captured photographs analyzed by the AI model; BMOS, Bedside Menu Ordering System; CBORD, proprietary foodservice management platform; DIMS, Dietary Intake Monitoring System; EHR, electronic health record; FRC, food-record chart; MI, Mobile Intake; MST, Malnutrition Screening Tool; NR, not reported; RGB-D, red-green-blue plus depth imaging; RFID, radio-frequency identification; WFR, weighed food record.

**Supplementary Table S4C. Detailed primary and supporting outcomes, findings and interpretation**

| **Report** | **Outcome domain** | **Outcomes assessed** | **Main quantitative/qualitative findings** | **Interpretation and key limitations** | **Evidence role** |
| --- | --- | --- | --- | --- | --- |
| van Bakel et al., 2024 | Plate waste; meal frequency; economic outcome | Meal frequency, plate waste and estimated food production/disposal cost | Meal frequency increased from 2.5 to 3.0-3.3 meals/patient/day. Mean plate waste fell from 81 g/served meal to 33-49 g during digital-system years (reported p values <0.001 to 0.010 by comparisons); associated costs decreased approximately 32%-56%. | A personalized digital multi-channel service was associated with more eating opportunities and less waste/cost. No patient-level intake or nutritional outcomes; repeated one-week measurements; no concurrent control and potential secular effects. | Direct outcome evidence: plate waste, meal frequency and cost |
| Svendsen et al., 2024 | Supporting usability evidence | Preferences, self-rated capability, interface problems and System Usability Scale (SUS) | 78.7% considered dish selection appropriate; 79.4% believed they could order independently. SUS improved from 59.2 (n=6) to 68.1 (n=4) after redesign. Login, date terminology, scrolling and meal-diary navigation were recurrent issues. | Patient involvement supported an acceptable menu and improved system usability, but further clinical-effectiveness testing is needed. Very small usability samples; excluded acutely ill/cognitively impaired or isolated patients; no intake or nutrition-status effect evaluation. | Supporting context: ordering-system usability |
| Terp et al., 2024 | Dietary intake and requirement attainment | Food availability, energy/protein intake, requirement attainment, feasibility and acceptance | Food availability increased 64% to 85% (p=0.004). Energy intake increased from 5,660±2,432 to 6,712±2,964 kJ (difference 1,052; 95% CI 111-1,993); protein 43±19 to 60±28 g (difference 17; 95% CI 9-26). Protein-requirement attainment rose 59±29% to 73±34% (p=0.013). Most could use/order, but few used intake monitoring independently. | Food'n'Go was feasible and associated with higher intake, while education and active intake registration required more support. Nonrandomized pre/post cohorts; single site; implementation and other temporal changes could confound outcomes. | Direct outcome evidence: dietary intake and requirement attainment |
| Wray et al., 2026 | Supporting delivery evidence | Feasibility, acceptability, communication, logistics and experience | Families and staff evaluated the model positively and supported scale-up. Important implementation issues included communication, ward validation, kitchen logistics and reliable delivery. Direct energy intake, nutritional status and waste effects were not quantified. | A web-based on-demand model was feasible in a specialist pediatric setting when supported by safety validation and operational redesign. Small short proof-of-concept, no control, limited quantitative outcome measurement and specialist setting. | Supporting context: on-demand delivery feasibility |
| Ellick et al., 2025 | Nutritional status and nutrition-care implementation | Service allocation, referral source, training/confidence, malnutrition prevalence, hospital-acquired malnutrition and incidents | Dietetic assistants completed 60% of inpatient dietetics occasions; 26% of assistant tasks were initiated from nursing screening. Most assistants reported adequate training/confidence. Malnutrition prevalence was 14%; no hospital-acquired malnutrition and no clinical incidents were identified. | Technology plus delegated workforce supported a feasible preliminary malnutrition model of care, but formal comparative effectiveness and economic evaluation are still required. Descriptive single-site implementation; variable denominators; no control/counterfactual; multiple simultaneous changes; patient experience and cost not formally assessed. | Direct descriptive/supporting evidence: malnutrition care pathway |
| Maunder et al., 2022 | Measurement validity and data completeness | Criterion validity, completion, energy/protein estimates and staff time | Stage 1: MI did not differ from WFR for energy (p=1.00); mean time MI 40.3 s vs FRC 174.1 s and WFR 371.4 s (p<0.001). Stage 2: incomplete records 2% MI vs 65% FRC (p<0.001); full-day data 93% MI vs 33% FRC. FRC reported 28% less daily energy and 20% less protein than MI; MI averaged 14.36 s/meal. | MI provided faster, more complete and more accurate routine intake information than paper charts. Stage 2 observers were trained and MI served as reference; short implementation; two hospitals; no patient outcome evaluation. | Measurement evidence: intake completeness and accuracy |
| Paulsen et al., 2020 | Nutritional status and nutrition care | Weight, nutritional-risk status at discharge, nutrition treatment/documentation, care plans and length of stay | No significant difference in weight change. Nutritional risk at discharge was 77% vs 94% (p=0.019); nutritional treatment was documented in 81% vs 57% (p=0.011); nutrition care plan 70% vs 16% (p<0.001). No beneficial length-of-stay effect was demonstrated. | MyFood improved nutrition-care processes and reduced the proportion at nutritional risk at discharge, without improving weight or length of stay. Unblinded single-centre RCT; modest sample; clinical outcomes may require longer exposure/follow-up. | Direct outcome evidence: nutritional status and nutrition care |
| Albaladejo et al., 2025 | Measurement validity: food consumption | Concordance and mean differences by food category | CCC: cereals/starches n=219, 0.957 (95% CI 0.945-0.965); meat/fish n=114, 0.845 (0.787-0.888); vegetables n=35, 0.767 (0.604-0.868). Mean prototype-manual differences: −12.01 g starch, +1.19 g meat/fish, −14.85 g vegetables. | Accuracy was high for starches and acceptable for meat/fish, but vegetables and tray/food heterogeneity require improvement before routine care. Pilot at one site; component/image observations not independent; limited dish categories; no patient-level nutrition outcomes. | Measurement evidence: food-consumption estimation |
| Liu et al., 2026 | Measurement validity: intake and leftovers | System Usability Scale and estimation error | SUS automatic device 63.2 vs manual tablet 56.0 (p=0.037). Total mean absolute error: visual 0.18, AI-manual 0.80, AI-automatic 1.72; RMSE: 0.56, 1.65 and 2.99, respectively. Visual assessment was most accurate, while the automatic device was more usable than manual tablet capture. | Automation improved usability but accuracy remained inferior to direct visual assessment for liquid foods. Only liquid foods, small nurse sample, repeated food observations and single setting; device accuracy requires refinement. | Measurement evidence: automated intake estimation and usability |
| Ryu et al., 2024 | Measurement validity: sodium intake | Estimated sodium, urinary sodium and correlation/modifiers | Adjusted AI sodium estimate 2,022.7 mg/day (IQR 1,396.2-2,564.4) vs 24-h urinary sodium 2,783 mg/day (IQR 1,955-4,922). Estimates were significantly correlated, but substantial disparity remained; diuretic use influenced the relationship. | AI imaging showed potential for sodium monitoring but cannot yet replace biological/reference assessment. High attrition (25/54 analyzed), small single-centre sample, diuretic/urine-collection effects and pilot design. | Measurement evidence: sodium-intake estimation |
| Tagi et al., 2024 | Measurement validity: dietary intake | RMSE, R², mean differences and correlations for nutrient intake | Energy RMSE: AI 8.12, image visual 8.49, direct visual 4.34. AI mean 71.7±23.9 kcal did not differ from weighing (p=0.82); image visual 75.5±23.2 (p<0.001) and direct visual 73.1±26.4 (p=0.007) differed. Spearman ρ ranged 0.89-0.97 for energy/macronutrients. | AI estimation was clinically feasible and more accurate than image-only visual estimation, but less accurate than direct visual assessment. Single-site liquid-food dataset; repeated components/meals; model performance may not generalize to solid/mixed dishes. | Measurement evidence: AI-assisted intake estimation |
| Munk et al., 2025 | Dietary intake; nutritional status; downstream outcomes | Energy/protein adequacy, screening/documentation, GLIM malnutrition, length of stay, readmission and mortality | Compared with 2019, ≥75% of energy needs was achieved by 70% vs 35% and ≥75% protein needs by 51% vs 24% (both p<0.001). Intake was documented for 42% and screening within 24 h for 25%. Among at-risk patients assessed, 74% were GLIM-malnourished. Median LOS 13 days (IQR 9-21), 30-day readmission 39%, and in-hospital/30-day mortality 22%; no significant outcome differences by risk group were reported. | The digital food concept was associated with improved intake adequacy, but screening/documentation and downstream clinical outcomes remained suboptimal. One-day cross-sectional design, historical comparator, incomplete intake data, selected longer-stay patients and no causal attribution. | Direct outcome evidence: intake adequacy and nutritional status |
| Olufson et al., 2024 | Supporting implementation evidence | Interprofessional practice, flexibility, visibility, person-centredness and workflow | Four themes: interaction between local context and technology; effects on staff participation; technology-enabled flexibility; and visibility/invisibility. Digitalization improved access and flexibility in some situations but could reduce customization and create silos. | Digital systems are not inherently enabling; local configuration, interoperability and work practices determine person-centred effects. Qualitative study in three units within one service; no quantitative patient outcomes; findings context dependent. | Supporting context: implementation and person-centred care |
| Barrington et al., 2018 | Dietary intake; plate waste | Ordered and consumed energy/protein, plate waste and meal experience | Ordered energy 8,683 vs 6,773 kJ (p=0.004) and protein 97 vs 82 g (p=0.023); consumed energy 6,457 vs 4,805 kJ and protein 73 vs 58 g (both p<0.001). Plate waste did not change. Improvements were reported for receiving ordered food (p<0.001) and choosing liked food (p=0.006). | Bedside terminal ordering improved dietary intake and selected meal-experience measures in oncology inpatients. Nonrandomized comparison; possible differences between cohorts; single specialist setting. | Direct outcome evidence: dietary intake and plate waste |
| Barsha et al., 2023 | Supporting service evidence | Overall foodservice satisfaction and model differences | Adjusted mean satisfaction: traditional 3.8217 (SE 0.0974), BMOS 4.2154 (0.0669), call-at-point-of-service 4.4781 (0.1351), room service 4.5079 (0.0697). Call-at-point-of-service and room service exceeded traditional; room service exceeded BMOS; BMOS vs traditional was not statistically significant. | Ordering closer to consumption and greater flexibility were associated with higher satisfaction. Nonrandomized models likely differed by site/time and patient mix; survey response selection; small call-at-point-of-service group. | Supporting context: foodservice satisfaction |
| Jamison et al., 1996 | Supporting service evidence | Interest, curiosity, convenience, availability, satisfaction, motivation, ease of use, appetite stimulation, estimated cost | Computerized menu preferred for interest 80%, curiosity 78%, convenience 77%, availability 76%, satisfaction 76%, and motivation 73%; ease 61% and appetite stimulation 57%. Estimated monthly cost: US$615 computerized vs US$2,093 printed; estimated payback 8.4 months. | Early evidence that an interactive hospital menu could be acceptable and potentially less costly than printed menus. Small single-site volunteer sample; brief exposure; questionnaire outcomes; order effects and clinical outcomes not evaluated. | Supporting context: menu acceptability and cost |
| Maunder et al., 2015 | Dietary intake | Energy intake, protein intake, patient preference, nutrition-assistant time and awareness | Energy intake 8,273 vs 6,273 kJ and protein intake 83 vs 66 g with the electronic system (both p<0.05). Eighty percent preferred the electronic bedside system; no additional total nutrition-assistant time was reported. | Bedside electronic spoken ordering was associated with higher energy and protein intake and strong patient preference. Nonrandomized pre/post design; possible temporal and case-mix confounding; single hospital. | Direct outcome evidence: dietary intake |
| McCray et al., 2018 | Dietary intake; plate waste; economic outcome | Energy/protein intake, goal attainment, plate waste, satisfaction, staff preference, time and food cost | Energy intake 6,232±2,523 vs 5,513±2,112 kJ (p=0.035); protein 78±36 vs 53±24 g (p<0.001); energy-goal attainment 73% vs 64% (p=0.021); protein-goal attainment 98% vs 70% (p<0.001). Plate waste fell from 30% to 26% (p<0.001); patient food costs decreased 19%; satisfaction was similar (74% vs 75% good/very good). | BMOS was associated with higher intake, less waste and lower food cost, although staff time per order increased. Retrospective nonrandomized audit; potential secular/confounding effects; one institution. | Direct outcome evidence: dietary intake, plate waste and cost |
| Fisher et al., 2023 | Nutritional-risk identification | Inter-rater agreement and association of dashboard risk with MST malnutrition risk | Inter-rater κ=0.69 (95% CI 0.65-0.72). Highest vs lowest dashboard-risk category was associated with MST risk (OR 1.93, 95% CI 1.17-3.19; p<0.01); after adding body weight, lower weight was the only significant predictor. | The dashboard showed potential for nutrition-risk surveillance, but incremental predictive value beyond weight requires further validation. Retrospective single-site study; no external validation; potential circularity/missing data and confounding by weight. | Direct/associational evidence: nutritional-risk identification |
| Paulsen et al., 2018 | Measurement validity: dietary intake | Agreement for energy/protein/fluid and food groups; usability | About half achieved ≥90% agreement with the reference for energy, protein and fluid on both days. Dinner had the lowest agreement. Bread/cereals were overestimated and fruit underestimated. Ninety percent found MyFood easy to use and 97% easy to navigate. | MyFood showed acceptable individual intake agreement for many patients and high usability, but food-group estimation needed refinement. Small two-department sample; only two recording days; partial self-report and limited generalizability. | Measurement evidence: dietary-intake recording |
| Roberts et al., 2017b | Supporting implementation evidence | Acceptability, perceived value, role boundaries, workflow and implementation requirements | Three themes: enacting patient participation, optimizing nutrition care, and implementation considerations. Staff were broadly positive but emphasized role clarity, workflow fit and adequate support. | Implementation success depends on explicit professional responsibilities and integration into routine work. Single-site voluntary sample; perceptions rather than measured service or patient outcomes. | Supporting context: staff workflow and implementation |
| Roberts et al., 2017a | Supporting implementation evidence | Usability, acceptance, perceived benefits and willingness to participate | Five themes: familiarity with technology affected confidence but was not essential; interface design was important; perceived benefit increased acceptance; technology enabled participation to differing degrees; participation depended on perceived importance of nutrition. | Patients generally viewed electronic foodservice technology positively, but design and individual support requirements influenced engagement. Small purposive single-site sample; short exposure; excluded patients unable to participate; no clinical-effectiveness outcomes. | Supporting context: patient usability and equity |
| Ofei et al., 2015 | Plate waste | Meal energy/protein consumption, plate waste, portion size and nutritional-risk interaction | Energy and protein consumed per meal represented up to approximately 15% of daily recommended intake. Larger portions were associated with more plate waste overall (β=0.008, SE=0.003, p=0.002) and at supper (β=0.008, SE=0.004, p=0.021); patients at nutritional risk generated more supper waste (p=0.001). | DIMS demonstrated that portion-size choice and nutritional status are relevant to hospital plate-waste generation. Observational single-site study; short measurement period; meal-level clustering and residual confounding possible. | Direct outcome evidence: plate waste |
| Ofei et al., 2019 | Measurement validity: intake and plate waste | Validity and inter-rater reliability for portion, energy and protein | Correlations with reference values were r=0.99 for energy and r=0.98 for protein. Inter-rater ICC was 0.88 for pre-meal portion estimation, 0.99 post-meal, and 0.99 for energy/protein; no systematic bias was identified. | The image-weighed technique was highly accurate in this small pilot dataset. Very small sample of meals; trained assessors; limited foods/site and uncertain routine-care generalizability. | Measurement evidence: intake and plate-waste estimation |
| Rattray et al., 2018 | Supporting safety evidence | Frequency, severity and location of therapeutic-diet meal errors | 69 errors among 906 meals (8%); 97% were classified as critical. Error occurrence by workflow source included menu planning 17%, assembly <1%, main-meal delivery 53%, mid-meal delivery 9% and patient-sourced consumption 33% (these are step-specific rates and are not additive). | Digital foodservice infrastructure does not eliminate diet errors; delivery and patient-sourced foods remain important safety gaps. Observational audit; no pre-digital comparator; one hospital; error classification dependent on audit procedures. | Supporting context: diet safety and workflow |

*Note.* Results are reproduced or closely paraphrased from the source reports; no effect sizes, p values, confidence intervals, or other statistics were recalculated. Unless otherwise specified, continuous data are reported as mean±SD or median (IQR), and the comparison order follows the wording in the table. Repeated observations remain at the analytical level used by the source. Evidence-role labels were assigned for this review; supporting outcomes are not interpreted as direct effects on dietary intake, nutritional status, or plate waste. *Abbreviations:* AI, artificial intelligence; AI-automatic, photographs captured by the automatic device and analyzed by the AI model; AI-manual, tablet-captured photographs analyzed by the AI model; BMOS, Bedside Menu Ordering System; CCC, concordance correlation coefficient; CI, confidence interval; DIMS, Dietary Intake Monitoring System; EER, estimated energy requirement; EPR, estimated protein requirement; FRC, food-record chart; GLIM, Global Leadership Initiative on Malnutrition; ICC, intraclass correlation coefficient; IQR, interquartile range; kcal, kilocalorie; kJ, kilojoule; LOS, length of stay; MI, Mobile Intake; MST, Malnutrition Screening Tool; NR, not reported; OR, odds ratio; R², coefficient of determination; RCT, randomized controlled trial; RMSE, root-mean-square error; SD, standard deviation; SE, standard error; SUS, System Usability Scale; WFR, weighed food record; β, regression coefficient; κ, Cohen’s kappa; ρ, Spearman rank correlation coefficient.

**Supplementary Table S5. Detailed quantitative and qualitative outcome data ordered by title domain**

| **Outcome domain** | **Report** | **Outcome/metric** | **Comparison or reference** | **Reported result** | **Statistical information** | **Direction/interpretive note** |
| --- | --- | --- | --- | --- | --- | --- |
| Dietary intake | van Bakel et al., 2024 | Meal frequency | Digital vs traditional | 3.0-3.3 vs 2.5 meals/patient/day | Descriptive/repeated comparison | More eating opportunities |
| Dietary intake | Terp et al., 2024 | Energy intake | Post vs pre | 6,712±2,964 vs 5,660±2,432 kJ; difference 1,052 (95% CI 111-1,993) | CI excludes zero | Higher |
| Dietary intake | Terp et al., 2024 | Protein intake | Post vs pre | 60±28 vs 43±19 g; difference 17 (95% CI 9-26) | CI excludes zero | Higher |
| Dietary intake | Terp et al., 2024 | Protein-requirement attainment | Post vs pre | 73±34% vs 59±29% | p=0.013 | Higher |
| Dietary intake | Munk et al., 2025 | ≥75% energy requirement | 2023 digital concept vs 2019 | 70% vs 35% | p<0.001 | Higher adequacy |
| Dietary intake | Munk et al., 2025 | ≥75% protein requirement | 2023 digital concept vs 2019 | 51% vs 24% | p<0.001 | Higher adequacy |
| Dietary intake | Barrington et al., 2018 | Ordered energy | Bedside system vs paper | 8,683 vs 6,773 kJ | p=0.004 | Higher ordered energy |
| Dietary intake | Barrington et al., 2018 | Consumed energy | Bedside system vs paper | 6,457 vs 4,805 kJ | p<0.001 | Higher intake |
| Dietary intake | Barrington et al., 2018 | Consumed protein | Bedside system vs paper | 73 vs 58 g | p<0.001 | Higher intake |
| Dietary intake | Maunder et al., 2015 | Energy intake | Electronic vs paper | 8,273 vs 6,273 kJ | p<0.05 | Higher with electronic system |
| Dietary intake | Maunder et al., 2015 | Protein intake | Electronic vs paper | 83 vs 66 g | p<0.05 | Higher with electronic system |
| Dietary intake | McCray et al., 2018 | Energy intake | BMOS vs traditional | 6,232±2,523 vs 5,513±2,112 kJ | p=0.035 | Higher |
| Dietary intake | McCray et al., 2018 | Protein intake | BMOS vs traditional | 78±36 vs 53±24 g | p<0.001 | Higher |
| Nutritional status and nutrition care | Ellick et al., 2025 | Malnutrition/safety | Hospital audit | 14% prevalence; no hospital-acquired malnutrition; no clinical incidents | Descriptive | Preliminary safety signal |
| Nutritional status and nutrition care | Paulsen et al., 2020 | Nutritional risk at discharge | MyFood vs control | 77% vs 94% | p=0.019 | Lower risk proportion |
| Nutritional status and nutrition care | Paulsen et al., 2020 | Nutrition treatment documented | MyFood vs control | 81% vs 57% | p=0.011 | Improved process |
| Nutritional status and nutrition care | Paulsen et al., 2020 | Nutrition care plan | MyFood vs control | 70% vs 16% | p<0.001 | Improved process |
| Nutritional status and nutrition care | Munk et al., 2025 | Process quality | Current audit | Intake documented 42%; screening within 24 h 25% | Descriptive | Residual implementation gap |
| Nutritional status and nutrition care | Fisher et al., 2023 | Malnutrition-risk association | Highest vs lowest dashboard risk | OR 1.93 (95% CI 1.17-3.19) | p<0.01 | Associated before weight adjustment |
| Nutritional status and nutrition care | Fisher et al., 2023 | Adjusted model | Dashboard plus weight | Lower weight was the only significant predictor | Multivariable model | Incremental value uncertain |
| Plate waste and economic outcomes | van Bakel et al., 2024 | Plate waste | Digital vs traditional | 33-49 vs 81 g/served meal | Reported p<0.001 to 0.010 across comparisons | Lower |
| Plate waste and economic outcomes | van Bakel et al., 2024 | Associated cost | Digital vs traditional | Approximately 32%-56% lower | Estimated production/disposal costs | Lower |
| Plate waste and economic outcomes | Barrington et al., 2018 | Plate waste | Bedside system vs paper | No material change reported | Not significant | No waste effect |
| Plate waste and economic outcomes | Jamison et al., 1996 | Estimated monthly cost | Computerized vs printed menu | US$615 vs US$2,093; estimated payback 8.4 months | Cost model, not observed economic trial | Potential cost saving |
| Plate waste and economic outcomes | McCray et al., 2018 | Plate waste | BMOS vs traditional | 26% vs 30% | p<0.001 | Lower |
| Plate waste and economic outcomes | McCray et al., 2018 | Patient food cost | Post- vs pre-implementation | 19% reduction | Descriptive economic analysis | Lower |
| Plate waste and economic outcomes | Ofei et al., 2015 | Plate waste and portion size | Larger vs smaller portions | Regression β=0.008, SE=0.003 | p=0.002 | Larger portions associated with more waste |
| Plate waste and economic outcomes | Ofei et al., 2015 | Supper waste | Larger portion | β=0.008, SE=0.004 | p=0.021 | More waste at supper |
| Plate waste and economic outcomes | Ofei et al., 2015 | Nutritional-risk subgroup | At risk vs not at risk at supper | At-risk patients generated more waste | p=0.001 | Risk status modified waste |
| Measurement validity and data quality | Maunder et al., 2022 | Energy agreement Stage 1 | MI vs WFR | No significant difference | p=1.00 | Criterion agreement |
| Measurement validity and data quality | Maunder et al., 2022 | Time Stage 1 | MI vs FRC vs WFR | 40.3 vs 174.1 vs 371.4 s | p<0.001 | MI fastest |
| Measurement validity and data quality | Maunder et al., 2022 | Incomplete records Stage 2 | MI vs FRC | 2% vs 65% | p<0.001 | MI more complete |
| Measurement validity and data quality | Maunder et al., 2022 | Daily energy/protein Stage 2 | FRC relative to MI | FRC 28% lower energy and 20% lower protein | Statistically significant comparisons reported | Paper charts underestimated |
| Measurement validity and data quality | Albaladejo et al., 2025 | Cereals/starches concordance | Prototype vs manual | CCC 0.957 (95% CI 0.945-0.965), n=219 | Lin CCC | Excellent |
| Measurement validity and data quality | Albaladejo et al., 2025 | Meat/fish concordance | Prototype vs manual | CCC 0.845 (95% CI 0.787-0.888), n=114 | Lin CCC | Good |
| Measurement validity and data quality | Albaladejo et al., 2025 | Vegetables concordance | Prototype vs manual | CCC 0.767 (95% CI 0.604-0.868), n=35 | Lin CCC | Lower/uncertain |
| Measurement validity and data quality | Liu et al., 2026 | Mean absolute error | Visual vs AI-manual vs AI-automatic | 0.18 vs 0.80 vs 1.72 | Comparative accuracy | Visual most accurate |
| Measurement validity and data quality | Liu et al., 2026 | RMSE | Visual vs AI-manual vs AI-automatic | 0.56 vs 1.65 vs 2.99 | Comparative accuracy | Automation accuracy lower |
| Measurement validity and data quality | Ryu et al., 2024 | Daily sodium | AI vs 24-h urinary sodium | 2,022.7 mg/day (IQR 1,396.2-2,564.4) vs 2,783 mg/day (IQR 1,955-4,922) | Significant correlation reported | Correlated but discrepant |
| Measurement validity and data quality | Ryu et al., 2024 | Completion | Enrolled vs analyzed | 54 vs 25 | Attrition | Feasibility limitation |
| Measurement validity and data quality | Tagi et al., 2024 | Energy RMSE | AI vs image visual vs direct visual | 8.12 vs 8.49 vs 4.34 | RMSE | AI better than image visual, worse than direct |
| Measurement validity and data quality | Tagi et al., 2024 | Mean energy estimate | AI vs weighing | 71.7±23.9 kcal | p=0.82 | No difference |
| Measurement validity and data quality | Tagi et al., 2024 | Nutrient correlations | All methods vs weighing | ρ=0.89-0.97 energy; 0.94-0.97 protein; 0.91-0.94 fat; 0.89-0.97 carbohydrate | Spearman correlations | High |
| Measurement validity and data quality | Fisher et al., 2023 | Inter-rater agreement | Dashboard intake coding | κ=0.69 (95% CI 0.65-0.72) | Kappa | Substantial/moderate agreement |
| Measurement validity and data quality | Paulsen et al., 2018 | Individual agreement | MyFood vs photo/partial weighing | About half had ≥90% agreement for energy, protein and fluid on both days | Descriptive individual agreement | Moderate agreement |
| Measurement validity and data quality | Ofei et al., 2019 | Energy validity | Image-weighed vs reference | r=0.99 | Correlation | Excellent |
| Measurement validity and data quality | Ofei et al., 2019 | Protein validity | Image-weighed vs reference | r=0.98 | Correlation | Excellent |
| Measurement validity and data quality | Ofei et al., 2019 | Inter-rater reliability | Pre/post portions and nutrients | ICC 0.88 pre-meal; 0.99 post-meal; 0.99 energy/protein | ICC | High, but small sample |
| Downstream clinical outcomes | Paulsen et al., 2020 | Weight/LOS | MyFood vs control | No significant beneficial effect | Not significant | No downstream effect demonstrated |
| Downstream clinical outcomes | Munk et al., 2025 | Downstream outcomes | Current cohort | Median LOS 13 days; 30-day readmission 39%; in-hospital/30-day mortality 22% | No significant differences by risk group reported | Clinical benefit unproven |
| Service, usability, implementation and safety | Svendsen et al., 2024 | Menu suitability | Questionnaire | 78.7% considered dish selection appropriate | Descriptive | Supports menu acceptability |
| Service, usability, implementation and safety | Svendsen et al., 2024 | Self-order capability | Questionnaire | 79.4% believed they could order independently | Descriptive | Potential access, with exclusions |
| Service, usability, implementation and safety | Svendsen et al., 2024 | Usability | Prototype round 1 vs 2 | SUS 59.2 vs 68.1 | Descriptive small samples | Improved after redesign |
| Service, usability, implementation and safety | Terp et al., 2024 | Food availability | Post vs pre | 85% vs 64% | p=0.004 | Improved |
| Service, usability, implementation and safety | Wray et al., 2026 | Feasibility, acceptability, communication, logistics and experience | Baseline foodservice experience before proof of concept | Families and staff evaluated the model positively and supported scale-up. Important implementation issues included communication, ward validation, kitchen logistics and reliable delivery. Direct energy intake, nutritional status and waste effects were not quantified. | Qualitative/descriptive as reported | A web-based on-demand model was feasible in a specialist pediatric setting when supported by safety validation and operational redesign. |
| Service, usability, implementation and safety | Ellick et al., 2025 | Delegated occasions | First implementation year | 60% of inpatient dietetics occasions completed by assistants | Descriptive | Substantial delegation |
| Service, usability, implementation and safety | Ellick et al., 2025 | Screening-initiated tasks | Assistant tasks | 26% initiated from nursing screening | Descriptive | Digital pathway generated work |
| Service, usability, implementation and safety | Liu et al., 2026 | Usability | Automatic vs manual tablet | SUS 63.2 vs 56.0 | p=0.037 | Automatic more usable |
| Service, usability, implementation and safety | Olufson et al., 2024 | Interprofessional practice, flexibility, visibility, person-centredness and workflow | Cross-unit contextual comparison; no formal intervention comparator | Four themes: interaction between local context and technology; effects on staff participation; technology-enabled flexibility; and visibility/invisibility. Digitalization improved access and flexibility in some situations but could reduce customization and create silos. | Qualitative/descriptive as reported | Digital systems are not inherently enabling; local configuration, interoperability and work practices determine person-centred effects. |
| Service, usability, implementation and safety | Barsha et al., 2023 | Adjusted satisfaction | Four ordering models | Traditional 3.8217; BMOS 4.2154; call-at-point-of-service 4.4781; room service 4.5079 | Model-based adjusted means | Greater flexibility generally higher |
| Service, usability, implementation and safety | Barsha et al., 2023 | Model contrasts | Ordering models | Call-at-point-of-service and room service > traditional; room service > BMOS; BMOS vs traditional not significant | Reported pairwise comparisons | Timing/flexibility important |
| Service, usability, implementation and safety | Jamison et al., 1996 | Patient preference domains | Computerized vs printed menu | Interest 80% vs 20%; curiosity 78% vs 22%; convenience 77% vs 23%; availability 76% vs 24%; satisfaction 76% vs 24%; motivation 73% vs 27% | First six domains reported as statistically significant in the source table | Favoured computerized menu |
| Service, usability, implementation and safety | Jamison et al., 1996 | Ease/appetite | Computerized vs printed menu | Ease 61% vs 39%; appetite stimulation 57% vs 43% | Not statistically significant | Positive trend only |
| Service, usability, implementation and safety | Maunder et al., 2015 | Preference | Electronic system | 80% preferred electronic bedside ordering | Descriptive | High acceptance |
| Service, usability, implementation and safety | Paulsen et al., 2018 | Usability | Patient report | 90% easy to use; 97% easy to navigate | Descriptive | High usability |
| Service, usability, implementation and safety | Roberts et al., 2017b | Acceptability, perceived value, role boundaries, workflow and implementation requirements | None | Three themes: enacting patient participation, optimizing nutrition care, and implementation considerations. Staff were broadly positive but emphasized role clarity, workflow fit and adequate support. | Qualitative/descriptive as reported | Implementation success depends on explicit professional responsibilities and integration into routine work. |
| Service, usability, implementation and safety | Roberts et al., 2017a | Usability, acceptance, perceived benefits and willingness to participate | None | Five themes: familiarity with technology affected confidence but was not essential; interface design was important; perceived benefit increased acceptance; technology enabled participation to differing degrees; participation depended on perceived importance of nutrition. | Qualitative/descriptive as reported | Patients generally viewed electronic foodservice technology positively, but design and individual support requirements influenced engagement. |
| Service, usability, implementation and safety | Rattray et al., 2018 | Therapeutic-diet meal errors | 906 meals | 69 errors (8%); 97% critical | Descriptive | Persistent safety risk |
| Service, usability, implementation and safety | Rattray et al., 2018 | Error source | Workflow classification | Step-specific error rates: main-meal delivery 53%; patient-sourced consumption 33%; menu planning 17%; mid-meal delivery 9%; assembly <1% | Descriptive; pathway categories | Delivery/patient sourcing dominant |

*Note.* Data are ordered by outcome domain and retain the unit of analysis reported by the source. No standardized effect size or additional significance test was calculated when the source did not provide one. Continuous data are reported as mean+/-SD or median (IQR) where specified; percentages and p values are reproduced as published and may reflect rounding. Direction/interpretive notes describe report-level observations only and do not imply causality, consistency, or certainty; interpretation should consider design, sample size, precision, comparator, cointerventions, and the appraisal in Supplementary Table S3. Abbreviations: AI, artificial intelligence; AI-automatic, photographs captured by the automatic device and analyzed by the AI model; AI-manual, tablet-captured photographs analyzed by the AI model; BMOS, Bedside Menu Ordering System; CCC, concordance correlation coefficient; CI, confidence interval; FRC, food-record chart; ICC, intraclass correlation coefficient; IQR, interquartile range; kcal, kilocalorie; kJ, kilojoule; LOS, length of stay; MI, Mobile Intake; MST, Malnutrition Screening Tool; OR, odds ratio; RMSE, root-mean-square error; SD, standard deviation; SE, standard error; SUS, System Usability Scale; WFR, weighed food record; beta, regression coefficient; kappa, Cohen's kappa; rho, Spearman rank correlation coefficient.

**Supplementary Table S6. Implementation, workflow and equity considerations supporting interpretation of the core outcomes**

| **Dimension** | **Barriers/risks** | **Facilitators/implementation responses** | **Supporting studies** | **Equity or evidence gap** |
| --- | --- | --- | --- | --- |
| Patient capability and digital access | Low confidence with unfamiliar technology; acute illness, cognitive, visual or motor limitations; some patients unable to self-order or record intake; very ill/isolated patients often excluded from usability work. | Simple image-supported interfaces; assisted ordering; repeated practice; alternative telephone/bedside channels; explicit explanation of clinical benefit. | Svendsen et al., 2024; Terp et al., 2024; Wray et al., 2026; Roberts et al., 2017a | Older, cognitively impaired, language-diverse and highly dependent patients remain underrepresented; assisted and nondigital alternatives should be preserved. |
| Interface and usability | Login difficulties, confusing date terminology, scrolling/navigation problems, touchscreen sensitivity, difficulty locating meal diaries; manual photo capture less usable. | Iterative patient co-design; think-aloud testing; simplified navigation; automatic image capture; visible order confirmation. | Svendsen et al., 2024; Liu et al., 2026; Jamison et al., 1996; Roberts et al., 2017a | Usability results may overestimate accessibility because severely ill and cognitively impaired patients were commonly excluded. |
| Workflow fit and staff workload | Paper charts incomplete; added bedside order-taking time; unclear responsibility for reviewing intake; digital tasks can duplicate existing work. | Capture at natural workflow points (bedside ordering or tray collection); automatic nutrient analysis; protocols; protected roles; delegation to trained assistants. | Ellick et al., 2025; Maunder et al., 2022; Maunder et al., 2015; McCray et al., 2018 | Time savings in measurement do not guarantee overall service savings; full time-and-motion and economic evaluations are limited. |
| Professional roles and interprofessional coordination | Digital systems can create silos, shift responsibility without agreement and reduce person-centred adaptation. | Role clarification; multidisciplinary governance; dietitian oversight; structured escalation; shared visibility of intake data. | Ellick et al., 2025; Olufson et al., 2024; Roberts et al., 2017b | Local organizational context strongly modifies impact; implementation models may not transfer unchanged between hospitals. |
| Data completeness and measurement accuracy | Manual records miss meals/items; AI errors vary by food category and presentation; image/weight tools may under- or overestimate specific components. | Integrated nutrient databases; pre/post imaging; weighed validation; staff training; automatic tray capture; category-specific algorithm calibration. | Maunder et al., 2022; Albaladejo et al., 2025; Liu et al., 2026; Ryu et al., 2024; Tagi et al., 2024; Paulsen et al., 2018; Ofei et al., 2019 | Most validation studies are single-site and use limited foods; external validation and clinically acceptable error thresholds are needed. |
| Interoperability and data visibility | Foodservice, EHR and nutrition systems may not exchange data; information can be visible to one group but invisible to another. | Dashboards, real-time intake reports, automated referrals and diet-order checks at point of service. | van Bakel et al., 2024; Ellick et al., 2025; Olufson et al., 2024; Fisher et al., 2023 | Integration was often incompletely reported; future studies should specify standards, data flow, latency and governance. |
| Clinical response and safety | Alerts can generate false positives; intake decline may not trigger action; digital ordering cannot prevent all therapeutic-diet errors. | Threshold refinement; automated referral plus human review; allergy/diet validation; dietitian escalation; audit feedback. | Wray et al., 2026; Ellick et al., 2025; Paulsen et al., 2020; Fisher et al., 2023; Rattray et al., 2018 | Downstream effects on complications, readmission and mortality remain sparsely tested; safety should not rely on automation alone. |
| Organizational and technical infrastructure | Device reliability, connectivity, kitchen capacity, delivery logistics, communication and change fatigue can undermine implementation. | Executive sponsorship; staged pilots; workflow redesign; kitchen-ward communication; staff training and feedback cycles. | Svendsen et al., 2024; Wray et al., 2026; Olufson et al., 2024; Roberts et al., 2017b | Implementation resources and maintenance costs were rarely fully quantified. |
| Sustainability and economic value | Waste reduction may be offset by infrastructure, labour or technology costs; environmental outcomes seldom measured beyond food waste. | Personalized/on-demand ordering, live demand data, smaller portions and more flexible eating opportunities. | van Bakel et al., 2024; Jamison et al., 1996; McCray et al., 2018 | Formal cost-effectiveness, lifecycle environmental assessment and long-term sustainability evidence are limited. |

*Note.* Dimensions were synthesized descriptively across included reports to explain implementation conditions, safety, workflow, and equity. Review-level categories were developed inductively from author-reported barriers and facilitators during primary charting and checked by the second reviewer; no predefined implementation framework was used for coding. Qualitative findings were integrated with quantitative evidence by juxtaposition within the relevant pathway domain, not by pooling or transformation. These dimensions are contextual determinants rather than pooled or causal effects, and a supporting study may contribute to more than one dimension. Evidence of underrepresentation or absent subgroup reporting is distinguished from demonstrated inequitable outcomes. Abbreviations: AI, artificial intelligence; EHR, electronic health record.
